# Large-scale recording of neuronal activity in freely-moving mice at cellular resolution

**DOI:** 10.1038/s41467-023-42083-y

**Published:** 2023-10-12

**Authors:** Aniruddha Das, Sarah Holden, Julie Borovicka, Jacob Icardi, Abigail O’Niel, Ariel Chaklai, Davina Patel, Rushik Patel, Stefanie Kaech Petrie, Jacob Raber, Hod Dana

**Affiliations:** 1 Department of Neurosciences, Lerner Research Institute, Cleveland Clinic Foundation, Cleveland, OH USA; 2 Department of Behavioral Neuroscience, Oregon Health and Science University, Portland, OR USA; 3 Jungers Center, Oregon Health and Science University, Portland, OR USA; 4 Departments of Neurology and Radiation Medicine, Division of Neuroscience, ONPRC, Oregon Health and Science University, Portland, OR USA; 5 Department of Molecular Medicine, Cleveland Clinic Lerner College of Medicine, School of Medicine, Case Western Reserve University, Cleveland, OH USA

**Keywords:** Visual system, Optical imaging

## Abstract

Current methods for recording large-scale neuronal activity from behaving mice at single-cell resolution require either fixing the mouse head under a microscope or attachment of a recording device to the animal’s skull. Both of these options significantly affect the animal behavior and hence also the recorded brain activity patterns. Here, we introduce a different method to acquire snapshots of single-cell cortical activity maps from freely-moving mice using a calcium sensor called CaMPARI. CaMPARI has a unique property of irreversibly changing its color from green to red inside active neurons when illuminated with 400 nm light. We capitalize on this property to demonstrate cortex-wide activity recording without any head fixation, tethering, or attachment of a miniaturized device to the mouse’s head. Multiple cortical regions were recorded while the mouse was performing a battery of behavioral and cognitive tests. We identified task-dependent activity patterns across motor and somatosensory cortices, with significant differences across sub-regions of the motor cortex and correlations across several activity patterns and task parameters. This CaMPARI-based recording method expands the capabilities of recording neuronal activity from freely-moving and behaving mice under minimally-restrictive experimental conditions and provides large-scale volumetric data that are currently not accessible otherwise.

## Abstract (web summary)

Single-cell resolution recording from behaving mice requires either head fixation or attachment of a miniaturized device which may alter behavior. Here, the authors present a new recording method without mechanical restrictions on mouse movement.

## Introduction

The mammalian brain processes sensory information using synchronized activity of brain-wide distributed neurons that are connected into local circuits^1–6^, which emphasizes the need for developing recording methods that are capable of capturing these complex activation patterns. To address this challenge, previous efforts have concentrated on improving the sensitivity of sensors to capture single-cell activity^7–12^ and enhancing the capability of recording systems to track neurons over large brain regions^13–17^. In parallel, scientific paradigms have shifted to analyzing neuronal activity in behaving animals while they process sensory cues to perform a task, with much of this work performed using the mouse as a model. This approach has enabled identifying the functional roles of specific cell types^18^, brain regions^4^, and/or projections between brain regions^1,6^, as well as determining how normal activity patterns are altered following a neurological condition^19,20^ or a model for neurodegenerative diseases^21,22^.

Currently, recordings from many neurons spanning multiple brain regions with single-cell resolution in behaving mice are mostly conducted using genetically-encoded calcium indicators (GECIs). These experimental paradigms include either monitoring of head-fixed mice using two-photon laser scanning microscopy (TPLSM)^23,24^, or attaching a miniaturized imaging device to the skull of a freely-moving mouse to record single-photon or two-photon fluorescence^25–27^. Importantly, both of these methods have inherent limitations. For example, TPLSM recording from behaving rodents requires head fixation of the mouse under a microscope, which may result in activation of different neuronal circuits compared to natural, freely-moving behaviors^28^. In addition, most state-of-the-art TPLSM systems are capable of recording from one large plane spanning up to several mm^2^, or from a few smaller, axially-shifted planes^15–17^. These microscopes are usually limited by the mechanics of laser scanning systems, which restrict the effective field-of-view (FOV) size that can be dynamically monitored. The acquired information is limited to the inside of this FOV, and so the activity of nearby neurons outside the FOV, even if they are labeled with a GECI, cannot be simultaneously detected. When brain activity is recorded using an implanted miniaturized imaging device, it allows head movement of the mouse during recording, but it puts a substantial weight on its skull. This additional weight may affect the mouse’s natural behaviors, and hence also the recorded neuronal activation patterns. In addition, the spatial resolution and volumetric recording capabilities are compromised compared to TPLSM recording.

Calcium-modulated photoactivatable ratiometric integrator (CaMPARI) is a calcium- and light-dependent fluorescent activity marker^29^, which may enable combining the relative simplicity of GECIs with TPLSM large-scale recording capabilities and free movement of the animal. Upon illumination with 400 nm light in a high-calcium environment, CaMPARI undergoes an irreversible conformational change, and its fluorescence emission changes from green to red in a process called photoconversion (PC). CaMPARI was previously demonstrated to label active neurons in the mouse visual cortex with red fluorescence based upon their tuning properties^29^, and a recent version of this sensor, CaMPARI2, exhibits a brighter signal and better contrast between the red and green components^30^.

CaMPARI’s unique calcium-dependent PC capability allowed us to design a distinct experimental paradigm where the experimental recording and signal readout processes are separated. In this study, we show that this paradigm shift facilitates neuronal activity recording over a larger brain volume than what has been possible with state-of-the-art TPLSM systems. Large-scale CaMPARI-based recording is conducted by shining a PC light over the animal and its experimental environment, which induces red fluorescence in active neurons in the mouse brain in a non-transient manner. The readout of the photoconverted CaMPARI fluorescence is conducted after the experiment is completed using a standard TPLSM system. This recording paradigm is fundamentally different than previous recordings with TPLSM systems, where the recording and readout processes occur simultaneously and cannot be separated. In this study, we reveal the advantages of CaMPARI-based recording for detecting activity from brain volumes larger than 6 mm^3^ with single-cell resolution. We validate the accuracy of the CaMPARI-based recording method by comparing the results to recordings with the widely-used GECI, jGCaMP7s^10^. We show functional differences between activity patterns of excitatory and parvalbumin-positive (PV-positive) inhibitory neurons when the mouse is presented with visual stimulation. Finally, we demonstrate the capability of the CaMPARI-based recording method to monitor single-neuron activity over a large cortical volume in freely-moving mice without any mechanical device attached to the mouse during the recording phase, in order to compare activity level patterns across five somatomotor cortical regions and to correlate these patterns with behavioral parameters as the mice perform a battery of behavioral tasks.

## Results

### Characterization of CaMPARI-based recording capabilities

**Fig. 1:**
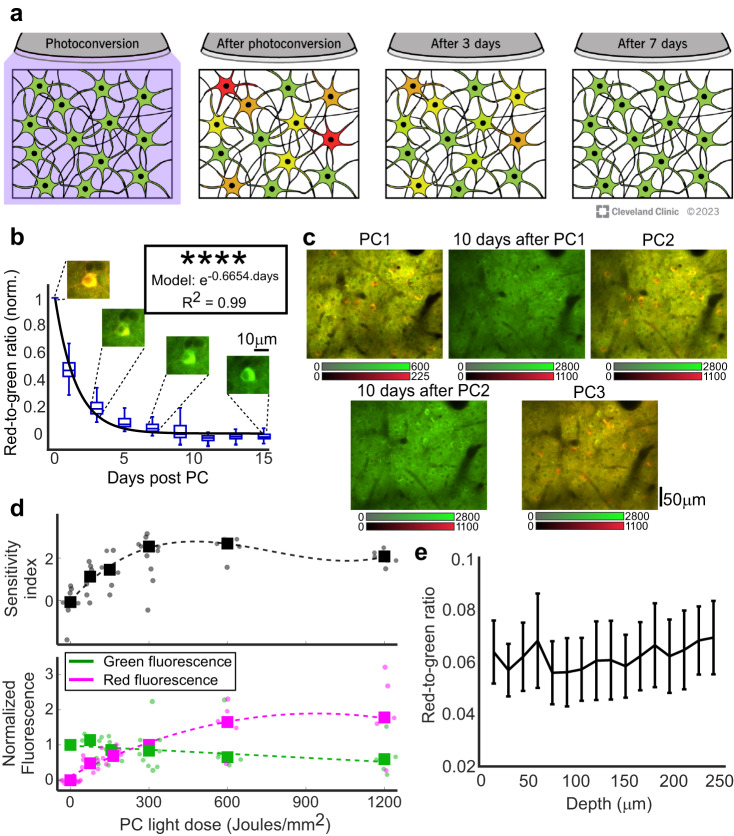
Characterization of CaMPARI-based recording. **a** Schematic illustration of the changes in CaMPARI green and red fluorescence following PC. **b** RGRs from photoconverted neurons (n = 73 cells, 2 mice) were normalized to each cell’s RGR level immediately after PC (Day 0) and were monitored for 15 days. Median values were fit with an exponential curve (t_1/2_ = 1.04 ± 0.076 days, mean ± SE; R^2^ = 0.99; for each boxplot, horizontal lines show medians, boxes show the 25th–75th percentiles, whisker length is the shorter of 1.5 times the 25th–75th range or the extreme data point). Example images of the same cell are presented above the respective days. **c** Example images of three repeated PCs in the same neurons (two repeated-recording experiments were conducted). **d** Upper panel, sensitivity index (d′) values for separating V1 and S1 activity levels during visual stimulation were increased with the light dose and reached an optimum near 300 J/mm^2^. Circular dots show the median values from single light dose recording from all identified cells, and the large squares are the mean across all single-experiment medians (n = 8 mice, 33 PC sessions, 3–9 sessions per mouse; 207–762 neurons/session, median of 480 neurons/session; mean data were fit with 3rd-order polynomial dashed line). Lower panel, median green and red fluorescence signals from all recorded cells were normalized to their pre-PC levels (green, fit with a 1st-order polynomial dashed line) and 300 J/mm^2^ light dose (red, fit with a 2nd-order polynomial magenta dashed line) values. The green signal gradually decreased, and the red signal increased with PC light dose (same data as in upper panel, n = 8). **e** The median RGR across all recorded cells in each FOV, obtained from all mice and brain regions, showed no apparent differences across depths down to 240 μm under the pia (n = 5 mice, data from 5 different motor and somatosensory regions, 2–110 cells/FOV, median = 17). The solid line connects the average of single FOV median RGRs and error bars show the standard error of mean. No significant differences were found between different recording depths (one-way ANOVA, p = 1.00). All statistical tests were two-tailed, source data are provided as a Source Data file. Reprinted with permission, Cleveland Clinic Foundation ©2023. All Rights Reserved.

Although the green-to-red PC was reported to be permanent at the single-protein level^29,30^, the photo-converted red-to-green ratio (RGR) in vivo decreased during the days following PC, such that ~97% of it decayed by one week (Fig. 1a and Supplementary Figs. 1, 2), presumably due to degradation of the red protein and production of new (green) protein. To calculate the rate of RGR decay, we longitudinally monitored V1 neurons (n = 73 from 2 mice) and measured the RGR after PC. The results were fit with an exponential decay model with a half-life of 1.04 days (R^2^ = 0.99; Fig. 1b). Multiple PCs of the same brain region and neurons were demonstrated by two additional recordings, separated by 10 days each, and yielding similar activity patterns (Fig. 1c). Therefore, we concluded that CaMPARI can be used multiple times for sequential recording sessions.

Next, we characterized CaMPARI’s capability to identify active brain regions as a function of the amount of PC light shined on the mouse brain (light dose). Mice were injected with an Adeno-associated virus (AAV) carrying the CaMPARI2 sequence into their primary visual and somatosensory cortices (V1 and S1, respectively; n = 8 mice). The medians across the RGR recorded from all neurons in each region were compared after illumination of PC light during the presentation of a drifting grating movie to the contralateral eye (see “Methods”). Light quanta of up to ~6 J/mm^2^ were delivered by illuminating a 5–7 mm-diameter cross-section around the craniotomy opening and using up to 200 mW PC light for 1 s, followed by 11 s without illumination to allow the tissue to cool down. No signs of thermal damage were found for any tested light doses up to 1150 J/mm^2^ (192 illumination cycles at full power; Supplementary Fig. 3). The average V1 RGR levels were higher than S1 for all tested light doses, and the sensitivity index (d′, see “Methods”) that quantifies the separation among the V1 and S1 RGR distributions increased with light dose to a peak at 300 J/mm^2^ (Fig. 1d, upper panel). As more PC light was used, the green CaMPARI fluorescence decreased down to 60% of its initial emission level and the red fluorescence increased (Fig. 1d, lower panel). Following these findings, a light dose range of 150–300 J/mm^2^ was selected for the subsequent visual stimulation experiments (with recorded neurons from either one or two hemispheres) to balance between sensitivity and PC illumination time. The optimal 300 J/mm^2^ dose levels could be easily achieved for illuminating one hemisphere but requires a 4-fold increase in illumination time when the two hemispheres are illuminated. Such a prolonged recording time may result in changes in the mouse condition during the recording, and therefore a lower light dose was used for these experiments.

In a separate set of experiments, the RGR values were measured at different tissue depths down to 240 µm under the pia. No apparent changes in RGR values were found for different depths (Fig. 1e, Supplementary Fig. 4), which suggests that the PC-based recording levels were not substantially biased by the tissue depth when Layer II/III neurons were monitored. These results were supported by measuring the RGR depth decay in neurons expressing mEOS, a calcium-insensitive photoconvertible protein that was used to develop CaMPARI^29^. Decay of 20–30% in RGR values was identified across Layer II/III depth, which presumably contributed a negligible fraction of the variability to the recorded data. A more substantial light attenuation was found for RGR levels across Layers II/III and V neurons (Supplementary Fig. 5).

### Simultaneous neuronal activity recording from multiple brain regions

**Fig. 2:**
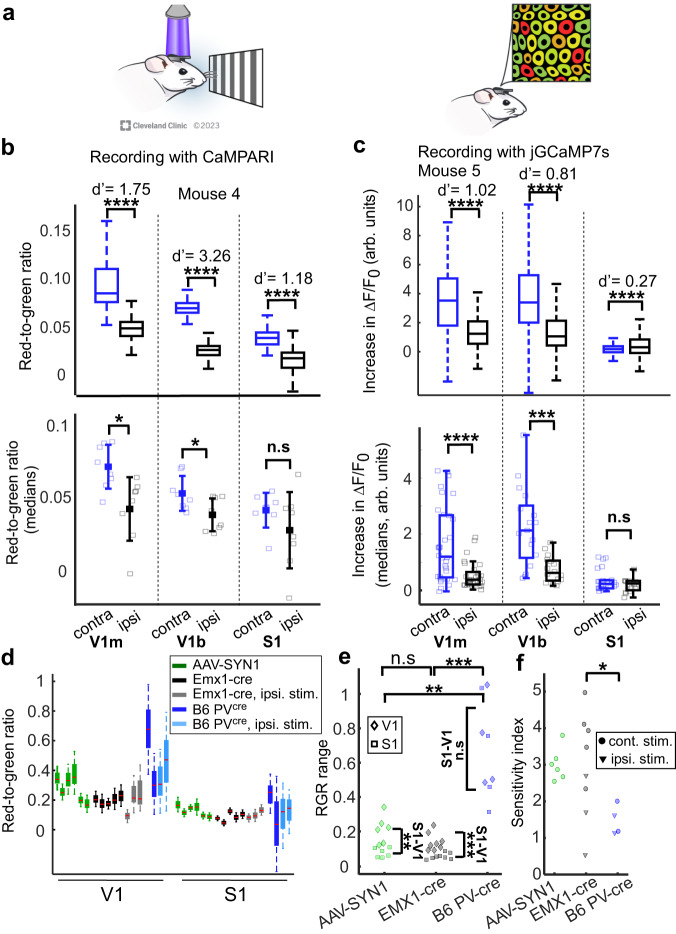
Simultaneous recording from multiple brain regions. **a** Schematic illustration of a visual-evoked activity recording experiment with CaMPARI. A drifting grating movie was presented to the mouse eye while PC light illuminated its two cortical hemispheres (left). Following PC, the imprinted cellular RGR was read using TPLSM (right). **b** Example data from a CaMPARI2-expressing mouse (top) showing simultaneous recording data from V1m, V1b, and S1 in both hemispheres (111–256 cells/region, median = 193; d′ values across the ipsilateral and contralateral regions are plotted; ****p < 0.0001, Wilcoxon Ranksum test for comparing RGR levels from all neurons in the contralateral vs. ipsilateral regions with Ranksum-statistic of 33,296, 50,599, and 68,231 for V1m, V1b, and S1, respectively). Activity levels are summarized by boxplots (horizontal lines show medians, boxes show the 25th–75th percentiles, whisker length is the shorter of 1.5 times the 25th–75th range or the extreme data point). Summary of the recorded activity levels measured with CaMPARI2 from n = 4 mice (bottom; each mouse was recorded twice with stimulation presented to each eye once; each rectangle shows the median RGR from 81–1455 cells/regions, median = 353; filled rectangles and error bars show the mean ± std; *p < 0.05; n.s not significant; paired t-test for comparing median values from contralateral vs. ipsilateral regions of the same mouse with p = 0.042, 0.027, and 0.166, and t-statistic of 2.48, 2.78, and 1.55 for V1m, V1b, and S1, respectively). **c** Example data from a jGCaMP7s-expressing mouse (top) showing similar distribution of increased fluorescence like (**b**) (229–898 cells/region, median=350). d′ values and significance levels are plotted (****p < 0.0001; Wilcoxon Ranksum test with p = 1.7*10^−36^, 6.2*10^−32^, and 4.8*10^−5^, and Ranksum-statistic of 98687, 88050, and 308589 for comparing contralateral vs. ipsilateral V1b, V1m, and S respectively). Summary of the recorded activity levels measured with jGCaMP7s (bottom) from n = 6 mice showed similar increases in fluorescence during stimulation like in CaMPARI2-expressing neurons (21–898 cells/regions, median = 161; ****p < 0.0001; ***p < 0.001); n.s not significant; two-sample t-tests with t-statistic of 4.18, 4.15, and 1.02 for comparing contralateral vs. ipsilateral V1m, V1b, and S1 from the same mice, respectively; boxplot representation as in (**b**). **d** RGR levels in V1 were higher than in S1 for excitatory and inhibitory CaMPARI2-expressing neurons. Among V1 neurons, RGR levels were highest in B6 PV^cre^ mice, than in AAV-SYN1 mice, and the lowest in the Emx1-cre mice (48.86 ± 26.7%, 27.33 ± 8%, and 19.55 ± 2.4%, respectively); mean ± std of the median RGRs across all recorded cells per mouse during contralateral eye stimulations; AAV-SYN1 group; n = 6 mice; green bars; Emx1-cre, n = 3 blue; B6 PV^cre^, n = 3, black. Each bar shows the 25–75 percentile range, red lines indicate the medians and whiskers span 0.722-fold of the interquartile range, which corresponds to an approximate range of the central 90% of normal distribution. Lighter color bars (gray and light blue) show the RGR for stimulation of the ipsilateral eye (data from 13,997 neurons recorded from 12 mice, 38–1235 neurons/regions, median = 256. Six AAV-SYN1 mice were recorded once with visual stimulation to the contralateral eye, and 3 Emx1-cre and 3 B6 PV^cre^ mice were recorded 1–3 times with visual stimulation presented to the contralateral and/or ipsilateral eye). **e** The 5–95 percentile range of RGR distribution of V1 neurons was significantly larger for B6 PV^cre^ neurons than for AAV-SYN1 neurons or Emx1-cre neurons (two-sample t-tests; p = 0.0001 with t-statistic = 5.96 and p = 0.003 with t-statistic = 4.33 for comparing the range from all V1 neurons in B6 PV^cre^ mice with Emx1-cre and AAV-SYN mice, respectively. The range of AAV-SYN neurons was non-significantly larger than of Emx1-cre neurons, p = 0.08, two-sampled t-test). In addition, the RGR range for V1 neurons was significantly larger than for S1 neurons in the AAV-SYN1 and Emx1-cre groups, but not in the B6 PV^cre^ group (0.21 ± 0.08 vs. 0.08 ± 0.03, 0.14 ± 0.06 vs. 0.06 ± 0.02, and 0.70 ± 0.27 vs. 0.64 ± 0.32, respectively, mean ± std.; paired t-tests; p = 0.0008 with t-statistic = 5.64, p = 0.003 with t-statistic=5.36, and p = 0.827, respectively; median V1 and S1 values from individual mice are shown in diamonds and rectangles, respectively; AAV-SYN1, data from 6 recordings from 6 mice; Emx1-cre, data from 8 recordings from 3 mice; B6 PV^cre^, data from 4 recordings from 3 mice). **f** The sensitivity index (d′) for separating the distribution of V1 and S1 RGRs was significantly higher for Emx1-cre neurons vs. B6 PV^cre^ neurons, and non-significantly higher for Emx1-cre vs. AAV-SYN1 neurons (two-sample t-tests, p = 0.034 with t-statistic = 2.90, and p = 0.12 for comparing contralateral stimulation, respectively. Emx1-cre, 5 measurements from 3 mice; AAV-SYN1, 6 measurements from 6 mice; B6 PV^cre^, 2 measurements from 2 mice). In addition, d′ was significantly higher for contralateral vs. ipsilateral eye stimulation for Emx1-cre neurons, but not for B6 PV^cre^ neurons (two-sample t-tests, p = 0.028 with t-statistic = 2.89, and p = 0.7, respectively). All statistical tests were two-tailed, source data are provided as a Source Data file. Reprinted with permission, Cleveland Clinic Foundation ©2023. All Rights Reserved.

Simultaneous volumetric recording over a large cortical area was accomplished by expressing CaMPARI2 in the monocular and binocular visual cortices (V1m and V1b, respectively) and the somatosensory cortices (S1) of the two hemispheres of mice (n = 4). A drifting grating movie was presented to either the right or left eye of lightly-anesthetized mice and was synchronized with PC illumination that illuminated both hemispheres and covered a brain volume of 6 mm^3^ (~10 mm^2^ of cortical surface for each hemispheric window, with detectable signal down to a depth of ~300 μm; 150 J/mm^2^ were used for PC to shorten the experiment duration). Once the PC recording was completed, the neuronal RGR was recorded using TPLSM (Fig. 2a). A control group (n = 6 mice) expressed jGCaMP7s in V1b, V1m, and S1 of one hemisphere and we recorded the fluorescence changes evoked by visual stimulation to each eye, as was previously done^7,10,31^. Both CaMPARI2- and jGCaMP7s-expressing neurons showed similar activity patterns, where visual regions were more active than somatosensory regions, and increased activity was detected in the contralateral compared to the ipsilateral visual regions (Fig. 2b, c, Supplementary Fig. 6). Notably, while CaMPARI-based data were recorded from all 6 brain regions simultaneously, jGCaMP7s-based data required a much longer recording process. Visual activity was recorded from 3–5 FOVs within each brain region sequentially, and therefore required combining recordings from 142 individual time points.

### Recording from genetically-targeted neuronal populations

Selective recording from genetically-targeted cellular populations was achieved by injecting cre-dependent CaMPARI2 AAV into the V1 and S1 regions of Emx1-cre^32^ or B6 PV^cre^ mice to express CaMPARI2 in either excitatory or PV-positive inhibitory neurons, respectively (n = 3 mice for each group). An additional group of C57BL/6 J mice (n = 6) were injected with AAV expressing CaMPARI2 under the human *synapsin1* promoter (AAV-SYN1) to express CaMPARI2 in both excitatory and inhibitory neurons. All mice were implanted with cranial windows on top of the left hemisphere. V1 and S1 activity was recorded from lightly-anesthetized mice during the presentation of a drifting grating movie to either the contralateral or ipsilateral eye using 300 J/mm^2^ light dose for PC. V1 RGR levels were higher than S1 for the three groups, with the highest levels measured in B6 PV^cre^ mice, then in AAV-SYN1 mice, and the lowest in the Emx1-cre mice, indicating different ranges of activity levels in these groups (Fig. 2d). Moreover, the range of RGRs measured from B6 PV^cre^ V1 neurons was significantly larger than from the other two groups (Fig. 2e). Interestingly, while the RGR range was significantly larger for V1 vs. S1 neurons for the AAV-SYN1 and Emx1-cre groups, there was no apparent difference for the B6 PV^cre^ group (Fig. 2e). When comparing the d′ for separating V1 and S1 RGR distributions across the different groups, we found that for contralateral eye stimulation, Emx1-cre had significantly higher d′ values than B6 PV^cre^ and non-significantly higher values than AAV-SYN1 (Fig. 2f). When comparing the d′ values for contralateral vs. ipsilateral eye stimulation, there were significantly higher d′ values for contralateral stimulation for the Emx1-cre mice and no apparent difference for the B6 PV^cre^ group.

### Large-scale volumetric recording of brain activity from freely-moving mice

**Fig. 3:**
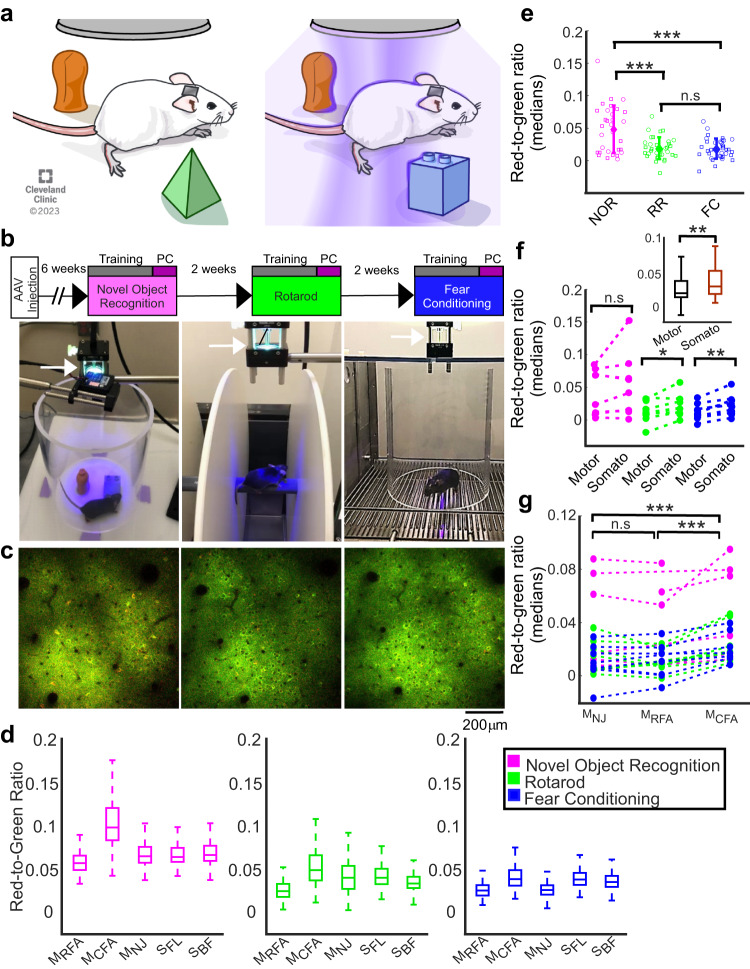
Recording large-scale, single-cell activity from freely-moving mice during behavioral and cognitive tasks. **a** Schematic illustration of the NOR experimental setup. A mouse is trained inside an arena (left) and the PC light is turned on when it performs the task (right). **b** Experimental timeline (top row) with images taken during NOR (left), RR (middle), and FC (right). The PC light source is indicated by white arrows. **c** Example post-PC images from the same brain region of the same mouse, which were acquired 24 h after the recording of each task two weeks apart. **d** RGR distribution in the five recorded cortical regions (same mouse as in (**c**); NOR: 204–819 cells/region, median 547; RR: 166–847, 495; FC: 229–1315, 874; one recording without repetitions; horizontal lines show medians, boxes show the 25th–75th percentiles, whisker length is the shorter of 1.5 times the 25^th^–75^th^ range or the extreme data point). **e** Median activity levels during NOR were significantly higher than those during RR and FC (***p < *0.001*, two-sample t-tests, p = 0.0001, 0.00003, and 0.81, with t-statistic = 4.18, 4.52, and 0.25 for comparing NOR vs. RR, NOR vs. FC, and FC vs. RR, respectively; n = 8 mice, squares - motor regions, circles – somatosensory regions; NOR: 42–906 cells/region, median 335; RR: 93–847, 271; FC: 85–1315, 315; n.s., non-significant; bold diamonds and error bars show the mean ± standard deviation). **f** Somatosensory regions were more active than motor regions across all tasks (inset; n = 8 mice; paired t-test; p = 0.0018 with t-statistic=3.52); boxplot representation as in (**d**). Across individual tasks, the increase was significant for RR and FC (paired t-test; p = 0.016 and 0.0012 with t-statistic=3.14 and 5.28, respectively*;* p = 0.25 for NOR; lines connect data from the same mouse). **g** Activity levels in M_CFA_ were significantly higher than in M_NJ_ and M_RFA_ (p = 0.0001 for both comparisons with t-statistic = 5.18 and 5.16, respectively; paired t-test from 16–19 pairs, n = 8 mice, 89–902 cells/region, median = 300; lines connect pairs or triplets of data from the same mouse). No significant differences were observed between M_NJ_ and M_RFA_ activity levels (p = 0.073). All statistical tests were two-tailed, source data are provided as a Source Data file. Reprinted with permission, Cleveland Clinic Foundation ©2023. All Rights Reserved.

Next, we moved to recording neuronal activity from freely-moving mice by expressing CaMPARI2 in 5 motor and somatosensory regions of the same hemisphere (motor caudal front limb, M_CFA_; motor rostral front limb, M_RFA_; motor neck-jaw, M_NJ_; somatosensory forelimbs, S_FL_; and somatosensory barrel field, S_BF_; n = 8 mice; see “Methods” for details). The same mice were trained and tested on three tasks (novel object recognition in the open field, NOR), rotarod (RR), and fear conditioning (FC), performing a new task every two weeks using arenas equipped with a PC light source (Fig. 3a). For all experiments, the mice were first trained for the particular behavioral task. Following the completion of the training phase, the next session was conducted with the PC light turned on to photoconvert cells during 15 min of recording (Fig. 3b, Supplementary movies 1–3, see “Methods” for details). A set of control experiments found no significant effects for the implantation of cranial window or illumination with 400 nm PC light on the tested performance parameters of the mice in the NOR, RR, and FC tasks (Supplementary Figs. 7–9). Readout sessions were conducted 24 h after recording (Fig. 3c and supplementary movie 4). RGR was measured from all identified neurons from the pial surface down to a depth of ~300 μm. Evaluating cellular RGRs across different cortical regions and tasks in the same mouse (Fig. 3d), as well as across mice (Supplementary Fig. 10), yielded a significant ~2.5-fold increase in NOR median activity levels compared to RR and FC (Fig. 3e). Somatosensory regions were more active than motor regions across the three behavioral tasks (Fig. 3f). When comparing activity across the different motor regions, M_CFA_ was significantly more active than M_RFA_ and M_NJ_, although M_CFA_ and M_RFA_ project to the same limb^33^ (Fig. 3g). There were no significant differences in activity levels between the two somatosensory regions S_BF_ and S_FL_ (Supplementary Fig. 11), or between cortical layers I and II/III. (Supplementary Fig. 12).

**Fig. 4:**
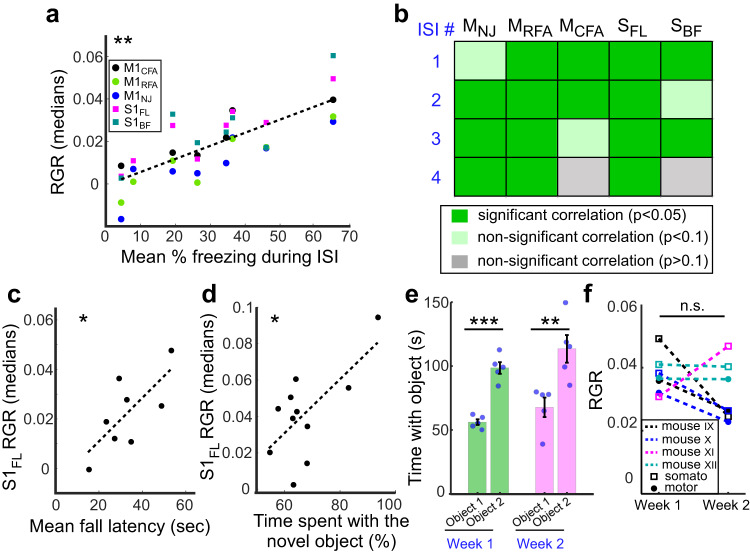
Correlation between brain activity recording and behavioral parameters and repeatability of CaMPARI recordings. **a, b** The percent freezing times during individual interstimulus intervals (ISIs) during FC for n = 8 mice were significantly correlated with the RGR levels in the majority of the recorded cortical regions. The average values from each mouse were significantly correlated with mean percent freezing during all ISIs (**a**); p = 0.0018, paired t-test, F-statistic vs. constant model = 28.5. When comparing individual brain regions and individual ISIs, 15/20 pairs showed significant correlations (**b**); p < 0.05, paired t-tests (see source data file). **c** The mean fall latency during day 1 of the RR training was significantly correlated with the RGR levels in S1_FL_ (p = 0.0424, paired t-test, F-statistic vs. constant model = 6.6; n = 8 mice). **d** The percent time spent with the novel object during the NOR task was significantly correlated with the RGR levels in S1_FL_ (p = 0.0233, paired t-test, F-statistic vs. constant model = 7.44; n = 11 mice). **e, f** Mice were tested and recorded with CaMPARI2 for two consecutive weeks on the NOR task with two new objects for each week. For both weeks, the exploration parameters of the new objects were similar (**e**), where mice spent significantly more time with the novel object than the known object (98.5 ± 10.2 vs. 56.1 ± 4.9 s, mean ± std., respectively for week 1; n = 5 mice, paired t-test, p = 0.0008 with t-statistic = 9.28. 113.4 ± 24.2 vs. 67.8 ± 17.1 s for exploring the novel vs. the known object on week 2; p = 0.005, t-statistic=5.58; same mice as in week 1). **f** RGR values were recorded every week and no significant changes were found between weeks 1 and 2 (p = 0.27, paired t-test, n = 4 mice, same mice as in (**e**), except one mouse where no signal could be recorded). All statistical tests were two-tailed, source data are provided as a Source Data file.

Interestingly, the averaged cortical activity measured during the FC memory test from all mice was correlated with the averaged interstimulus intervals (ISIs) during fear memory learning on day 1 of the task (Fig. 4a; n = 8 mice). Moreover, the activity of individual brain regions showed significant correlations with at least two out of the four individual ISIs (Fig. 4b), suggesting that activity in the somatomotor cortex during the memory test reflects aspects of the fear learning process. Among the recorded brain regions, S1_FL_ showed correlation with all ISIs. Interestingly, we saw a similar pattern for the rotarod test, where S1_FL_ was correlated with the mean fall latency during the learning phase of the test (Fig. 4c; n = 8 mice), and for the NOR test, where S1_FL_ activity was correlated with the time spent with the novel object (Fig. 4d; n = 11 mice).

Finally, we tested the reproducibility of brain activity recording in another set of experiments. Mice were tested in the novel object recognition task for two subsequent weeks using two new objects every week, and their brain activity was recorded during the novel object recognition phase (see “Methods”). For both weeks, the mice showed the expected preference towards the novel object, but no apparent difference in behavioral parameters like the total or the percent of time spent with the novel object, or the discrimination index (Fig. 4e, Supplementary Fig. 13). Similarly, no significant differences in the median RGRs of the somatosensory or motor regions were identified (Fig. 4f).

## Discussion

This work introduces a new method for acquiring simultaneous volumetric neuronal activity patterns from freely-moving rodents without mounting the animal’s head under a microscope, tethering it, or attaching a miniaturized imaging device to its skull. This method enables large-scale recording across multiple brain regions at single-cell resolution. Therefore, this paradigm overcomes the respective limitations of TPLSM-based recording (optimized for planar data acquisition from head-mounted rodents) and miniaturized device-based recording methods (challenges with volumetric acquisition, image quality, and FOV size). This method is especially beneficial for studying cortex-wide activity patterns in behaving mice, where minimal restrictions on the animal’s naturalistic performance are required^34^. Importantly, CaMPARI-based recording is performed simultaneously across the entire PC-illuminated volume, and therefore may highlight brain regions and/or specific cell types that are active during specific behaviors. This large-scale mapping may serve as the first step to identify active brain regions (Fig. 2c and Fig. 3d–g) followed by a more detailed study of dynamic action potential firing patterns using either CaMPARI’s dynamic recording capabilities^29,30,35^ or by expressing a more sensitive GECI in the target brain region(s). CaMPARI-based recording is also compatible with mapping of cortex-wide activity patterns in response to different stimulations, including sensory, chemogenetic, or optogenetic^36–39^.

This study demonstrates the benefits of the CaMPARI-based recording method for several applications. First, previous works characterized the relationship between firing of action potentials and PC rate in cultured neurons^29,30^. However, the use of CaMPARI in rodents was mostly limited for acute PC and downstream analysis following tissue sectioning. The return of the RGR values to their baseline level within ~7 days facilitates same-animal longitudinal monitoring experiments, as we demonstrated in this study (Fig. 3). Second, as was previously shown by widefield one-photon microscopy in either head-fixed mice^40^ or rats implanted with a miniaturized microscope^12^, when a visual stimulation is presented to one eye, the visual-evoked response is usually stronger in the contralateral V1, but is also apparent on the ipsilateral V1. Our data are consistent with these findings (Fig. 2b, c) and add the additional advantage of acquiring single-cell resolution data, which is not achievable using single-photon techniques. Third, in addition to single-cell resolution data, the presented method records simultaneously from thousands of genetically-targeted cells, a feature which is generally not achievable using electrophysiological recordings including high-density electrodes. We demonstrate this feature by conducting a comparative study across inhibitory and excitatory neurons in V1 and S1 and identifying cell-type-specific differences in their activity patterns (Fig. 2d–f). Interestingly, our data suggest that similar to the visual tuning properties of excitatory and inhibitory neurons in the visual system^41^, the excitatory neurons in V1 and S1 differentiate in their response to the stimulus, while the PV-positive neurons show a broader and partially-overlapping range of activity levels. Notably, measurements of the sensitivity indices showed a sharper separation of V1 and S1 population activity based on recording excitatory neurons only (Fig. 2f). However, even the PV-positive neurons, which exhibited reduced sensitivity compared to the other recorded groups, showed higher RGRs in V1, suggesting that all recorded cell types present a qualitatively similar activation pattern. Future applications of targeted recording during behavior may probe the role of these and other cell types in the neuronal circuitry underlying the performance of specific tasks or to highlight the spatiotemporal structure of their activity pattern across multiple brain regions. Finally, we demonstrate recording from freely-moving mice under what we consider as minimally-restrictive conditions, compared to the standards in the field today. No mechanical device or tethering were attached to the mice during their training and recording, and the only interventions they experienced were the craniotomy surgery and virus injection, which are currently unavoidable.

Three behavioral tasks were selected for the study: NOR, RR, and FC, to study three different types of behaviors and the associated neuronal circuitry. The NOR represents a somatosensory-centered task with a memory component, the RR is a motor-centric task, and the FC task is memory-centered, which includes dominant sub-cortical components and the somato-motor regions are considered less central. We selected to record brain activity from primary somato-motor regions to rely on the existing literature on the relationship between sensory stimuli and the neuronal activity in these regions. Notably, all mice were tested in a certain order: first NOR, then RR, and finally FC. The rationale was that the fear involved with FC may affect any following task, and thus this task was last. During RR, mice may fall and potentially be injured, so we placed this task after the NOR, which was considered to be safe. Due to these considerations, there was no randomization of the mouse testing order. Although two sequential tests of mice for the NOR task (Fig. 4e, f, Supplementary Fig. 13) and up to three times for the visual stimulation experiments (data not shown) showed no significant change of the results, such an effect cannot be fully excluded.

Unlike most existing recording methods, CaMPARI-based recording allows separation of the recording and readout processes. This separation allows conducting the recording by shining the PC light over an entire arena in which the rodent is contained, and to simultaneously record from all brain regions that are exposed to the PC light. Importantly, the ability to use CaMPARI-based recording for same-animal longitudinal monitoring differentiates this approach from commonly-used immediate early gene-based methods that require sacrificing the animal in order to read the activity data^42,43^. Interestingly, a recent work presented an in vivo method for identifying active cells based upon transgenic expression of the immediate early gene Fos in hippocampal cells^44^. We note that this method also allows conceptually similar separation of the recording and readout processes. However, since it relies upon a more complex, and partially unknown, mechanism that links neuronal firing to expression of immediate early genes, its current implementation enables classification of cells to Fos-high and Fos-low groups, and its temporal resolution is measured in hours. CaMPARI’s PC rate was shown to have an approximately linear relationship with low-to-medium firing rates of action potentials by neurons^29,30^, and our data (Figs. 2b, d, 3e–g) support the recording of different activity levels in different brain regions and across different tasks and cell types. We also note that future works may incorporate the use of the recently-published reversible CaMPARI (rsCaMPARI)^45^, which will allow erasing the CaMPARI activity signal immediately after the readout session, and therefore eliminate the current restriction of CaMPARI2 with regard to the recording interval period.

The presented method is limited to acquiring snapshots of large-scale activity patterns. The CaMPARI2 sensor, which was used in this study, requires a relatively prolonged PC illumination time for achieving high-quality recording in freely-moving mice (up to 15 min in this study). Shortening the recording session will further enhance the method’s capability to monitor brain activity and highlight co-active brain regions across shorter time scales. Such improvements may be achieved by using the earlier generation of the CaMPARI sensor, CaMPARI1^29^, which was recently shown to have better PC properties in vivo than CaMPARI2^35^, or by developing a new generation of the CaMPARI construct to address this specific challenge. In addition, in this study, we have expressed CaMPARI via intracranial AAV injection in specific cortical areas. Thus, we were limited to a finite number of brain areas that could be monitored. The cortex-wide recording capabilities of CaMPARI could be maximized by using systemic AAV injection^46,47^ or by developing a transgenic CaMPARI mouse line. Both of these options would enable expressing CaMPARI over most of the mouse cortex, which would achieve access to approximately 1 million neurons without substantially changing the presented recording protocol^48^.

Finally, we established recording from the same mice performing three different behavioral and cognitive tasks, and show changes in brain activity patterns across tasks and brain regions, with correlations between activity and behavioral patterns (Figs. 3 and 4). This type of data demonstrates that CaMPARI-based recording facilitates longitudinal in vivo neuronal activity studies during minimally-restricted behaviors in the same animals. With the recent increase in interest in studying large-scale brain activity patterns, and specifically the characteristics of distributed neuronal circuits^49,50^, the presented method adds unique capabilities and complements the tools that are available to the neuroscience community.

## Methods

All experimental and surgical procedures were performed following the set guidelines and protocols approved by the Lerner Research Institute (LRI) and Oregon Health & Science University (OHSU) Institutional Animal Care and Use Committees (IACUCs) and Institutional Biosafety Committees (IBCs) and were consistent with the ARRIVE guidelines. Mice were group-housed in standard vivarium conditions until the start of the study. The vivarium was maintained at 20–22 °C, 30–70% humidity and food (in LRI facility: Teklad 2918 regular diet, Envigo; in OHSU facility: PicoLab Rodent Diet 20, no. 5053; PMI Nutrition International) and water were available *ad libitum*. Lights were kept on a 12-h light/12-h dark cycle, and experiments were conducted during the light time.

### Surgical procedure and virus injection

For recording visual-evoked activity (Fig. 2), 8–12-week-old C57BL6/J, Emx1-Cre, and B6 PV^cre^ mice (10 males and 12 females) were anesthetized using isoflurane (3% for induction, 1.5% during the surgery) and placed on a heating pad. Each mouse was injected with local pain medication (bupivacaine 0.5%) and the skull bone above either the two cortical hemispheres (n = 4 mice) or the left hemisphere (rest of the mice) was exposed. A 3 × 5 mm^2^ craniotomy was drilled (Omnidrill35, World Precision Instruments) over an area covering the monocular and binocular primary visual (V1m and V1b, respectively) and primary somatosensory cortices (S1) in one or both hemispheres. AAV solution expressing the CaMPARI2 or jGCaMP7s sensor under the human *synapsin* promoter (SYN1-NES-his-CaMPARI2-WPRE-SV40, Addgene catalog number 101060, SYN1-jGCaMP7s-WPRE, Addgene catalog number 104487) was injected into two locations, separated by ~600 μm, in each cortical region (50 nL of ~1 × 10^12^ GC/mL solution, 3 injection depths per location, 200 μm, 400 μm, and 600 μm under the pia) using an automated injection pump (Fusion 200 touch Syringe Pump, Chemyx, and Micro-2T, WPI) and a pulled and beveled micropipette (P-1000 and BV-10, respectively, Sutter Instruments). Injection coordinates were chosen according to the mouse brain atlas^51^ 2:.2 mm lateral and 0.2 mm anterior to Lambda (V1m), 2.8 lateral and 0.2 mm anterior to Lambda (V1b), and 2.5 mm lateral and 3.4 anterior to Lambda (S1). For Emx1-cre^32^ (JAX catalog # 005628) and B6 PV^cre^ (JAX catalog #017320) mice, a cre-dependent AAV was used (AAV-PHP.N-SYN1-flex-CaMPARI2, Canadian Neurophotonics Platform Viral Vector Core)^47^. Cortex buffer^52^ was used consistently to keep the brain wet during the time of surgery and injections. Following the viral injection, a cranial window (two glued layers of rectangular glass, Tower Optical Corporation) was placed carefully (two cranial windows in the case of craniotomy in both hemispheres), and a custom-made metallic head bar was attached using dental cement (Contemporary Ortho-Jet, Lang Dental). Animals were injected with Buprenorphine (0.1 mg/kg) and Ketoprofen (5 mg/kg, immediately, 24, and 48 h after the surgery) for post-operative care and were allowed a minimal recovery time of 3 weeks before the start of experiments.

For recording activity from awake, behaving mice (Figs. 3 and 4), a similar surgical procedure was used with 8-12 week-old C57BL6/J mice (10 males and 3 females), but the AAV was injected into 5 locations into the mouse left motor and somatosensory cortices, according to microstimulation study^33^ and mouse brain atlas coordinates^51^: (1) 1.25/2 mm lateral/anterior to Bregma (rostral forelimb area of the motor cortex, M_RFA_); (2) 1.25/0 mm (caudal forelimb area of the motor cortex, M_CFA_); (3) 2/1.5 mm (neck and jaw regions of the motor cortex, M_NJ_); (4) 2/0 mm (forelimb region of the primary somatosensory cortex, S_FL_); and (5) 2.5/−1.75 mm (somatosensory barrel field cortex, close to the border with the trunk region, S_BF_). SYN1-NES-his-CaMPARI2-WPRE-SV40 solution (40 nL) was injected at depths of 250 μm, 500 μm, and 750 μm in each location. For control experiments (Supplementary Figs. 7–9), similar procedures were done, where saline was injected instead of AAV solution for 15 8-12 week-old C57BL6/J male mice. Finally, 12 naïve 8-12 week-old C57BL6/J male mice were also tested (Supplementary Figs. 7–9) without any surgical intervention.

### Recording of visual-evoked activity

Mice were lightly anesthetized (0.5% isoflurane), held on a 37 °C heating pad, and injected with Chlorprothixene Hydrochloride (IM, 30 μL of 0.33 mg/mL solution, Santa Cruz). PC of the CaMPARI2 signal started at least 30 min after the Chlorprothixene Hydrochloride injection, and after verifying that the mouse was responsive to pain but not voluntarily moving. The visual stimulation was presented to the mouse’s right or left eye and generated using the psychophysical toolbox^53,54^ in MATLAB (Mathworks) on an LCD monitor (30 × 36 cm^2^ display, located 15 cm in from of the mouse right eye, tilted 45° with respect to the nose line, and covered with a blue plexiglass to minimize contamination into the recording channels) that subtended an angle of ±50° horizontally and ±45° vertically. The visual stimulus consisted of a drifting grating moving in 1 of 8 directions for 4 s, followed by 8 s of gray display. This stimulation cycle was repeated 5 times. PC light was delivered for 1 s during the presentation of the drifting grating, 1.5 s after the grating appeared, using an X-Cite Fire or Xylis lamps (Excelitas) and a 400/40 nm bandpass filter (Brightline, Semrock) with up to 120 (Fire) or 200 (Xylis) mW output at the sample plane. For recording of visual-evoked activity, the PC light covered either a ~12 mm-diameter circle that included the two cranial windows in it, with an intensity of ~0.9 mW/mm^2^, or a 5–7 mm-diameter circle that covered one hemisphere with intensities of up to ~6 mW/mm^2^. After PC was completed, CaMPARI2 signal was recorded using a two-photon microscope with resonant/galvo scanners (Bergamo II, Thorlabs) with 1040 nm excitation light (Insight X3, Spectra-Physics). Images were acquired using ThorImage software (Thorlabs) with 15 frames per second and 1024 × 1024 pixels covering an area of 585 × 585 μm^2^ of layer II/III neurons. Green and red CaMPARI2 signals were recorded simultaneously (525/50 nm and 607/70 nm filters, respectively, separated by a 562 nm dichroic filter, Semrock) using 2 GaAsP PMTs (PMT2100, Thorlabs). For measuring the decay of red CaMPARI signal over time, the same V1 neurons were monitored immediately after the PC and over the subsequent 15 days.

For measuring visual-evoked fluorescence changes with jGCaMP7s^10^, the same TPLSM system described above was used with acquisition of 30 frames per second, 512 × 512 pixels, and the same FOV size. The same drifting grating movie (with 4 s of drifting grating followed by 4 s of gray display) was presented to either the right or the left eye of the mice, and activity was measured from V1b, V1m, and S1 regions of the left cortical hemisphere to sequentially acquire ipsilateral and contralateral activity data.

### Recording of cellular activity from freely-moving animals

All mice were injected with AAV expressing CaMPARI2 and implanted with cranial windows over their left hemisphere as described above and were tested in 4 separated cohorts. Eight of these mice were used for the experiments shown in Fig. 3, and additional 5 were used for the repeated NOR recordings and correlation between brain activity and behavior shown in Fig. 4. Following the craniotomy, mice were given 7 days for recovery and were shipped from the LRI to the OHSU, where they were given an additional 3 weeks for quarantine and recovery. N = 8 mice were then randomly divided into 2 groups. Each week, one group was trained for 2–3 days on one of three behavioral tasks (see below). The additional 5 mice were tested for exploratory behavior, measures of anxiety, and object recognition (details below) twice for two following weeks, to identify the recording reproducibility (data from the first week’s recording was also grouped with the data recorded from the previous 8 mice). The mouse cranial window was illuminated with PC light during the last trial of a given behavioral test. A broadband light source (X-Cite Xylis, Excelitas) with a 400/40 nm bandpass filter (FBH400-40, Thorlabs) and lightly focused by a 100 mm achromat lens (AC254-100-A, Thorlabs) was placed 20 cm above the arena to illuminate a circular cross-section of 15.25 cm in diameter in which the CaMPARI PC occurred. The light intensity was 330–485 mW, or ~2.65 mW/cm^2^ (~250-fold lower than the maximal intensity used with head-fixed mice). Mice were placed inside a plastic enclosure (16.5 cm diameter; TAP plastics) on matte white plastic flooring to keep them inside the illuminated region, and 15 min of PC illumination were used for each recording to elicit sufficient PC signal (based upon preliminary experiments we conducted with other mice to calculate the required light dose and duration).

Activity readouts were acquired using two-photon microscopy 24 h after the PC during the behavioral and cognitive tests. Mice were anesthetized with isoflurane (4% induction, 1.5% maintenance) and put on a heating pad with core body temperature monitored by a rectal thermometer. Imaging was conducted with a Zeiss LSM 7 multiphoton microscope (Zeiss instruments), which utilizes a femtosecond-pulsed Ti: Sapphire laser (Chameleon Ultra II, Coherent), two BiG.2 GaAsP detectors, and Zen imaging software (Zeiss). The laser was tuned to 1040 nm excitation wavelength at 16 mW when recording from the brain surface and up to 24 mW when imaging 300 µm under the pia. Distilled water was placed on the cranial window to image with a 20×/1.0 water immersion objective (Zeiss, 421452-9880). We acquired volumetric data of layers I and II/III neurons (z-stack of ~100 images typically from the brain surface, 512 × 512 pixels, 425 µm FOV size, 3 μm step size between adjacent images) with green and red channels (500–550 nm and 575–610 nm, respectively, with a 560 nm dichroic filter) from all identified CaMPARI2-injected regions in all animals.

### Behavioral testing

(1) Exploratory behavior, measures of anxiety, and object recognition were assessed in an open field for a total of 4 consecutive days. Days 1 and 2 included exposure to the open field without objects for 5 min/day. Introduction of two identical objects occurred on day 3, and day 4 consisted of replacing one of the familiar objects with a novel one and 400 nm illumination over the entire arena. Both days with objects (3–4) consisted of 15-min trials. Behavior was recorded with Ethovision 15 XT software. Camera and PC light-guide suspensions over the arena were built with Thorlabs mechanical component. For the mice that were tested and recorded twice, the objects were replaced between the first and second tests.

(2) Sensorimotor performance on the rotarod was tested for three consecutive days containing three trials each. Days 1 and 2 consisted of a standard rotarod protocol, with a starting speed of 5 RPM that was increased by 1 RPM every 3 s. On the third day, parameters were adjusted to a starting speed of 4 RPM, a maximum speed of 12 RPM, and an increase of 1 RPM every 60 s. This ensured that the animals remained on the rod for 5 min per trial (3× trials per day) and allowed for full 15-min illumination while on the rod. Experimenters noted the speed at which the rod was turning when the animal fell off and the duration of time that the animal was able to stay on the rod before falling.

(3) Contextual fear memory over two days was tested using a Med Associates mouse fear conditioning system for use with optogenetics (MED-VFC-OPTO-USB-M, Med Associates). During the training day, the animal was placed in the plastic arena (described above) that was located inside a white LED-lit (100 lux) fear conditioning chamber with a metal grid floor. Animals were habituated to the arena for a 300-s baseline period, followed by a 2-s, 0.7 mA foot shock, administered a total of 4 times at 60-s intervals. The following day, animals were placed in the same plastic enclosure within the fear conditioning chamber and contextual fear memory recall was tested for 15 min under illumination. Movement in the chamber and the percentage of freezing were automatically determined by the Med Associates VideoFreeze automated scoring system (MED-SYST-VFC-USB2, Med Associates).

### Data analysis

Data analysis was performed using custom MATLAB scripts. For segmenting the neuronal cell bodies we used the CellPose software^55^, or in cases where CellPose yielded low quality outcomes, a semi-automatic algorithm^7^ that allows the experimenter to manually detect the somata location. We compared the performance of the two segmentation methods on identical data and found that they produced similar results (Supplementary Fig. 14). For each recording day, we calculated and corrected the effect of dark current values for the green and red PMT channels by recording images for each channel with the same exposure parameters used for the experiments, but with the laser turned off. Since CaMPARI’s green signal penetrates the red channel^29^, we calculated and corrected for the red-to-green contamination ratio by recording CaMPARI images before PC (pre-PC) and measuring the contamination ratio. Finally, when mice with dual-hemispheric windows were recorded, the angle of each cortical window was different with respect to the microscope’s optical axis, and we measured a weak component of red channel auto-fluorescence for each hemisphere that was corrected by finding the intercept of the linear regression line of the green and red signals from cells from the same hemisphere before PC (Supplementary Fig. 15). Following these corrections, we calculated the post-PC red-to-green ratio (RGR) for all cells in a brain region and used the median value for comparisons across regions. We note that for the tested B6 PV^cre^ mice the green expression level of CaMPARI2 was dimmer than for the other mouse lines (Emx1-cre and C57BL6/J) and in addition to the green fluorescence of CaMPARI2, there was also some red dots that appeared inside and outside the somata before PC. In order to eliminate bias to our RGR calculations, we updated our acquisition and analysis scheme for this mouse line only to capture images of the same FOVs and cells before and after PC for all brain regions. We estimated green-to-red contamination from the pre-PC data and corrected the values for each mouse. Then, we calculated the RGR by subtracting the post-PC red by the pre-PC red values for each cell and dividing the outcome by the same cell’s post-PC green fluorescence.

We calculated the sensitivity index (d′) to measure the separation between visual and somatosensory cortical regions following the formula mentioned below:

1 $$\,{{{{{{\rm{d}}}}}}}^{{\prime} }=\frac{({{{{{\rm{mea}}}}}}{{{{{{\rm{n}}}}}}}_{{{{{{\rm{RG}}}}}}{{{{{{\rm{R}}}}}}}_{{{{{{\rm{visual}}}}}}}}-{{{{{\rm{mea}}}}}}{{{{{{\rm{n}}}}}}}_{{{{{{\rm{RG}}}}}}{{{{{{\rm{R}}}}}}}_{{{{{{\rm{somatosensory}}}}}}}})}{\surd (0.5\left(\right.{{{{{\rm{varianc}}}}}}{{{{{{\rm{e}}}}}}}_{{{{{{\rm{RG}}}}}}{{{{{{\rm{R}}}}}}}_{{{{{{\rm{visual}}}}}}}}+{{{{{\rm{varianc}}}}}}{{{{{{\rm{e}}}}}}}_{{{{{{\rm{RG}}}}}}{{{{{{\rm{R}}}}}}}_{{{{{{\rm{somatosensory}}}}}}}})}$$

For light dose calculations, we consecutively photoconverted the same brain region several times on the same day and measured the RGR from all cells after each PC. The light dose was calculated as the total light intensity for each PC event divided by the illumination cross-section and multiplied by the illumination time. For all recording experiments, which occurred over several years and using different light sources and in different locations, we measured the light intensity and changed experimental parameters, such as the PC illumination dimension and the number of illumination cycles, to maintain the same light dose values. For recording of two freely-moving mice, where the light source intensity decreased by 30% due to mechanical failure, we adjusted the measured RGR values, assuming linearity of the red and green signals in this range, as we found in Fig. 1d. For analyzing the recorded images from freely-moving mice, we first averaged every 3 adjacent images (spanning 6 μm in the z-axis) and skipped the next 2 images, in order to generate a set of images with minimal overlap of cells. We performed the same signal corrections as described above and calculated the RGR for all recorded cells and the median values for comparing across brain regions, mice, and tasks.

For calculating visual-evoked fluorescence changes measured by jGCaMP7s, we registered the recorded movie for small brain movements^56^. Then we CellPose to segment the cell bodies and we integrated the fluorescence changes (F_resp_) above the baseline level (F_base_) during all appearances of the drifting grating stimulation for each recorded neuron by using the formula:

2 $$[{{{{{\rm{sum}}}}}}({{{{{\rm{F}}}}}}_{{{{{\rm{resp}}}}}}/2)\,{{{-}}}\,{{{{{\rm{sum}}}}}}({{{{{\rm{F}}}}}}_{{{{{\rm{base}}}}}})]$$

F_resp_ was measured over the 4 s where the visual stimuli were presented, and F_base_ was measured over the 2 s before the appearance of the respective drifting grating.

All mice were injected in all brain regions during the craniotomy surgery (either V1b, V1m, and S1, or the five somatomotor regions). When imaging the CaMPARI fluorescence from the mice, the experimenter identified the brain regions according to a map of the injection locations. In case the expression level was too low in a specific site, this brain region was not imaged, and in case the expression was identified as poor during the analysis, the region was excluded. All other brain regions were included in the analysis.

Statistical comparisons included one-way ANOVA tests, Wilcoxon Ranksum tests, and paired and two-sample t-tests. All tests were two-tailed and no additional tests for normality or corrections for multiple comparisons were incorporated.

### Reporting summary

Further information on research design is available in the Nature Portfolio Reporting Summary linked to this article.

### Supplementary information

Supplementary Information

Description of Additional Supplementary Files

Supplementary Movie 1

Supplementary Movie 2

Supplementary Movie 3

Supplementary Movie 4

Reporting Summary

### Source data

Source Data

## Data Availability

All relevant data are included as part of the supplementary information or have been deposited in the FigShare database under accession code 10.6084/m9.figshare.24065970. Source data are provided with this paper.

## Code availability

Custom MATLAB scripts that were used for data analysis have been deposited in the FigShare database under accession code 10.6084/m9.figshare.24076851.

## References

[1] Inagaki HK (2022). A midbrain-thalamus-cortex circuit reorganizes cortical dynamics to initiate movement. Cell.

[2] Steinmetz NA, Zatka-Haas P, Carandini M, Harris KD (2019). Distributed coding of choice, action and engagement across the mouse brain. Nature.

[3] Makino H (2017). Transformation of cortex-wide emergent properties during motor learning. Neuron.

[4] Chen T-W, Li N, Daie K, Svoboda K (2017). A map of anticipatory activity in mouse motor cortex. Neuron.

[5] Scott BB (2018). Imaging cortical dynamics in GCaMP transgenic rats with a head-mounted widefield macroscope. Neuron.

[6] Chen JL (2015). Pathway-specific reorganization of projection neurons in somatosensory cortex during learning. Nat. Neurosci..

[7] Chen TW (2013). Ultrasensitive fluorescent proteins for imaging neuronal activity. Nature.

[8] Inoue M (2015). Rational design of a high-affinity, fast, red calcium indicator R-CaMP2. Nat. Methods.

[9] Dana, H. et al. Sensitive red protein calcium indicators for imaging neural activity. *Elife* **5**, e12727 (2016). 10.7554/eLife.12727 PMC4846379 27011354

[10] Dana H (2019). High-performance calcium sensors for imaging activity in neuronal populations and microcompartments. Nat. Methods.

[11] Inoue M (2019). Rational engineering of XCaMPs, a multicolor GECI suite for in vivo imaging of complex brain circuit dynamics. Cell.

[12] Zhang, Y. et al. Fast and sensitive GCaMP calcium indicators for imaging neural populations. *Nature* **615**, 884–891 (2023). 10.1038/s41586-023-05828-9 PMC10060165 36922596

[13] Tsai PS (2015). Ultra-large field-of-view two-photon microscopy. Opt. Express.

[14] Lu R (2020). Rapid mesoscale volumetric imaging of neural activity with synaptic resolution. Nat. Methods.

[15] Ota K (2021). Fast, cell-resolution, contiguous-wide two-photon imaging to reveal functional network architectures across multi-modal cortical areas. Neuron.

[16] Sofroniew, N. J., Flickinger, D., King, J. & Svoboda, K. A large field of view two-photon mesoscope with subcellular resolution for in vivo imaging. *Elife* **5**. 10.7554/eLife.14472 (2016). 10.7554/eLife.14472 PMC4951199 27300105

[17] Stirman JN, Smith IT, Kudenov MW, Smith SL (2016). Wide field-of-view, multi-region, two-photon imaging of neuronal activity in the mammalian brain. Nat Biotechnol..

[18] Allen WE (2017). Global representations of goal-directed behavior in distinct cell types of mouse neocortex. Neuron.

[19] Chornyy S (2021). Cellular-resolution monitoring of ischemic stroke pathologies in the rat cortex. Biomed. Opt. Express.

[20] Chornyy, S. et al. Longitudinal in vivo monitoring of axonal degeneration after brain injury. *Cell Rep. Methods*10.1016/j.crmeth.2023.100481 (2023). 10.1016/j.crmeth.2023.100481 PMC10261926 37323578

[21] Korzhova V (2021). Long-term dynamics of aberrant neuronal activity in awake Alzheimer’s disease transgenic mice. Commun. Biol..

[22] Das A (2020). Reversible loss of hippocampal function in a mouse model of demyelination/remyelination. Front. Cell. Neurosci..

[23] Sofroniew NJ, Vlasov YA, Hires SA, Freeman J, Svoboda K (2015). Neural coding in barrel cortex during whisker-guided locomotion. eLife.

[24] Huber D (2012). Multiple dynamic representations in the motor cortex during sensorimotor learning. Nature.

[25] Guo, C. et al. Miniscope-LFOV: A large-field-of-view, single-cell-resolution, miniature microscope for wired and wire-free imaging of neural dynamics in freely behaving animals. *Sci. Adv.* **9**, eadg3918 (2023). 10.1126/sciadv.adg3918 PMC10121160 37083539

[26] Skocek O (2018). High-speed volumetric imaging of neuronal activity in freely moving rodents. Nat. Methods.

[27] Zong W (2022). Large-scale two-photon calcium imaging in freely moving mice. Cell.

[28] Whishaw IQ (2017). Organization of the reach and grasp in head-fixed vs freely-moving mice provides support for multiple motor channel theory of neocortical organization. Exp. Brain Res..

[29] Fosque BF (2015). Neural circuits. Labeling of active neural circuits in vivo with designed calcium integrators. Science.

[30] Moeyaert B (2018). Improved methods for marking active neuron populations. Nat. Commun..

[31] Akerboom J (2012). Optimization of a GCaMP calcium indicator for neural activity imaging. J. Neurosci..

[32] Gorski JA (2002). Cortical excitatory neurons and glia, but not GABAergic neurons, are produced in the Emx1-expressing lineage. J. Neurosci..

[33] Tennant KA (2011). The organization of the forelimb representation of the C57BL/6 mouse motor cortex as defined by intracortical microstimulation and cytoarchitecture. Cereb. Cortex.

[34] Miller CT (2022). Natural behavior is the language of the brain. Curr. Biol..

[35] Das, A. et al. Enhanced detection sensitivity of neuronal activity patterns using CaMPARI1 vs. CaMPARI2. *Front. Neurosci.* **16**. 10.3389/fnins.2022.1055554 (2023). 10.3389/fnins.2022.1055554 PMC9871923 36704000

[36] Boyden ES, Zhang F, Bamberg E, Nagel G, Deisseroth K (2005). Millisecond-timescale, genetically targeted optical control of neural activity. Nat. Neurosci..

[37] Armbruster BN, Li X, Pausch MH, Herlitze S, Roth BL (2007). Evolving the lock to fit the key to create a family of G protein-coupled receptors potently activated by an inert ligand. Proc. Natl Acad. Sci. USA.

[38] Roth BL (2016). DREADDs for neuroscientists. Neuron.

[39] Yizhar O (2011). Optogenetics in neural systems. Neuron.

[40] Musall S, Kaufman MT, Juavinett AL, Gluf S, Churchland AK (2019). Single-trial neural dynamics are dominated by richly varied movements. Nat. Neurosci..

[41] Niell CM, Scanziani M (2021). How cortical circuits implement cortical computations: mouse visual cortex as a model. Annu. Rev. Neurosci..

[42] Guzowski JF (2005). Mapping behaviorally relevant neural circuits with immediate-early gene expression. Curr. Opin. Neurobiol..

[43] Renier N (2016). Mapping of brain activity by automated volume analysis of immediate early genes. Cell.

[44] Pettit NL, Yap E-L, Greenberg ME, Harvey CD (2022). Fos ensembles encode and shape stable spatial maps in the hippocampus. Nature.

[45] Sha F, Abdelfattah AS, Patel R, Schreiter ER (2020). Erasable labeling of neuronal activity using a reversible calcium marker. eLife.

[46] Chan KY (2017). Engineered AAVs for efficient noninvasive gene delivery to the central and peripheral nervous systems. Nat. Neurosci..

[47] Ravindra Kumar S (2020). Multiplexed cre-dependent selection yields systemic AAVs for targeting distinct brain cell types. Nat. Methods.

[48] Kim TH (2016). Long-Term optical access to an estimated one million neurons in the live mouse cortex. Cell Rep..

[49] Insel TR, Landis SC, Collins FS (2013). The NIH brain initiative. Science.

[50] Jorgenson LA (2015). The BRAIN Initiative: developing technology to catalyse neuroscience discovery. Philos. Trans. R. Soc. B Biol. Sci..

[51] Paxinos, G. & Franklin, K. B. *Paxinos and Franklin’s the Mouse Brain in Stereotaxic Coordinates* (Academic Press, 2019).

[52] Holtmaat A (2012). Imaging neocortical neurons through a chronic cranial window. Cold Spring Harb. Protoc..

[53] Brainard DH (1997). The Psychophysics Toolbox. Spat. Vis..

[54] Pelli DG (1997). The VideoToolbox software for visual psychophysics: transforming numbers into movies. Spat. Vis..

[55] Stringer C, Wang T, Michaelos M, Pachitariu M (2021). Cellpose: a generalist algorithm for cellular segmentation. Nat. Methods.

[56] Thevenaz P, Ruttimann UE, Unser M (1998). A pyramid approach to subpixel registration based on intensity. IEEE Trans. Image Process..

