# Supplementary material for: Large-scale recording of neuronal activity in freely-moving mice at cellular resolution
Source: Nat Commun. 2023 Oct 12;14:6399. doi: 10.1038/s41467-023-42083-y (PMC10570384; doi:10.1038/s41467-023-42083-y)
Supplement: Supplementary file 1 — Supplementary Information [file 41467_2023_42083_MOESM1_ESM.pdf]

Large-scale recording of neuronal activity in freely-moving mice at cellular resolution

All statistical tests were two-tailed with no corrections for multiple comparisons. Source data are provided as a Source Data file.

Supplementary Figures

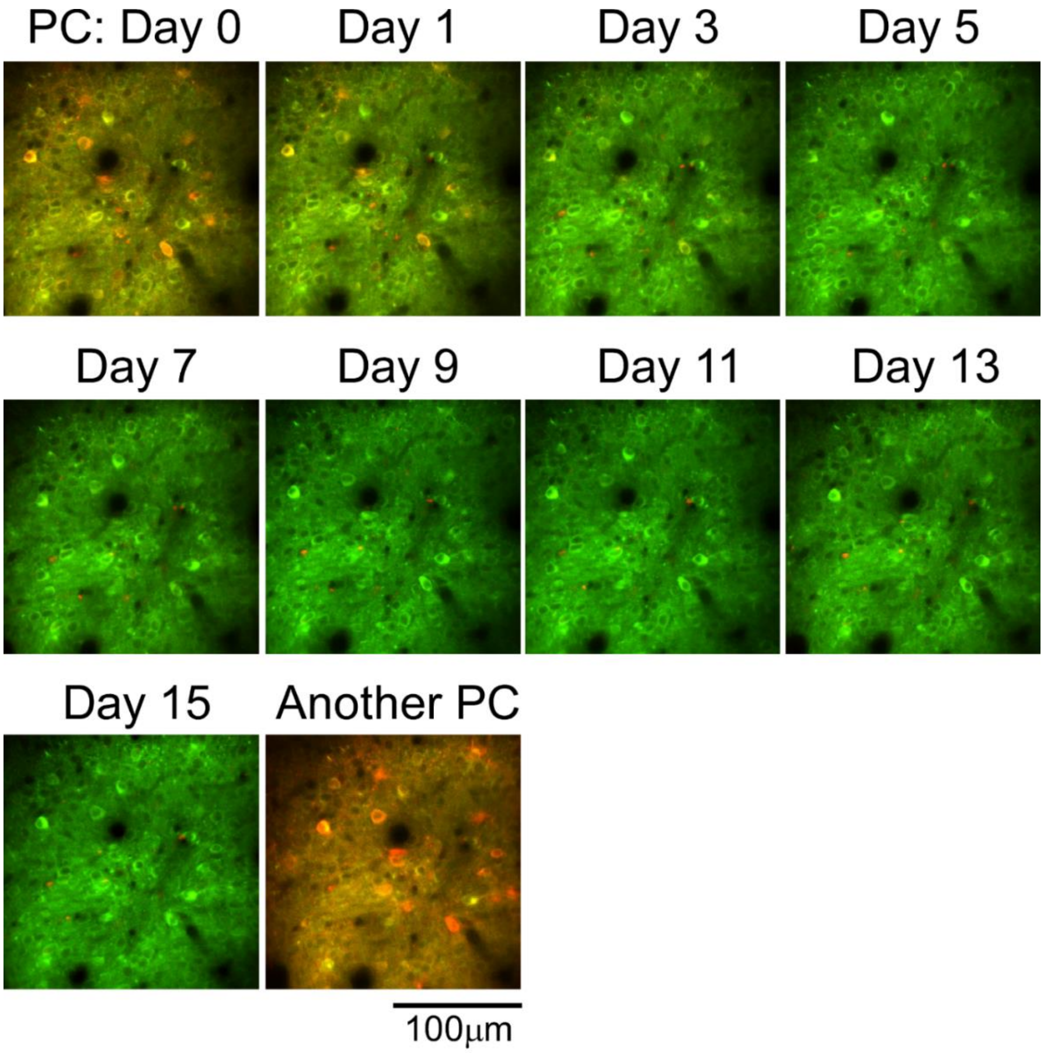

Supplementary Figure 1. Decay of RGR over time. Images of the same FOV were captured immediately after PC during visual stimulation (Day 0) and over the following 15 days. After the last recording session on Day 15, CaMPARI was again photoconverted using the same visual stimulation (“Another PC”). Repeated PC experiments were conducted in two different mice with similar results.

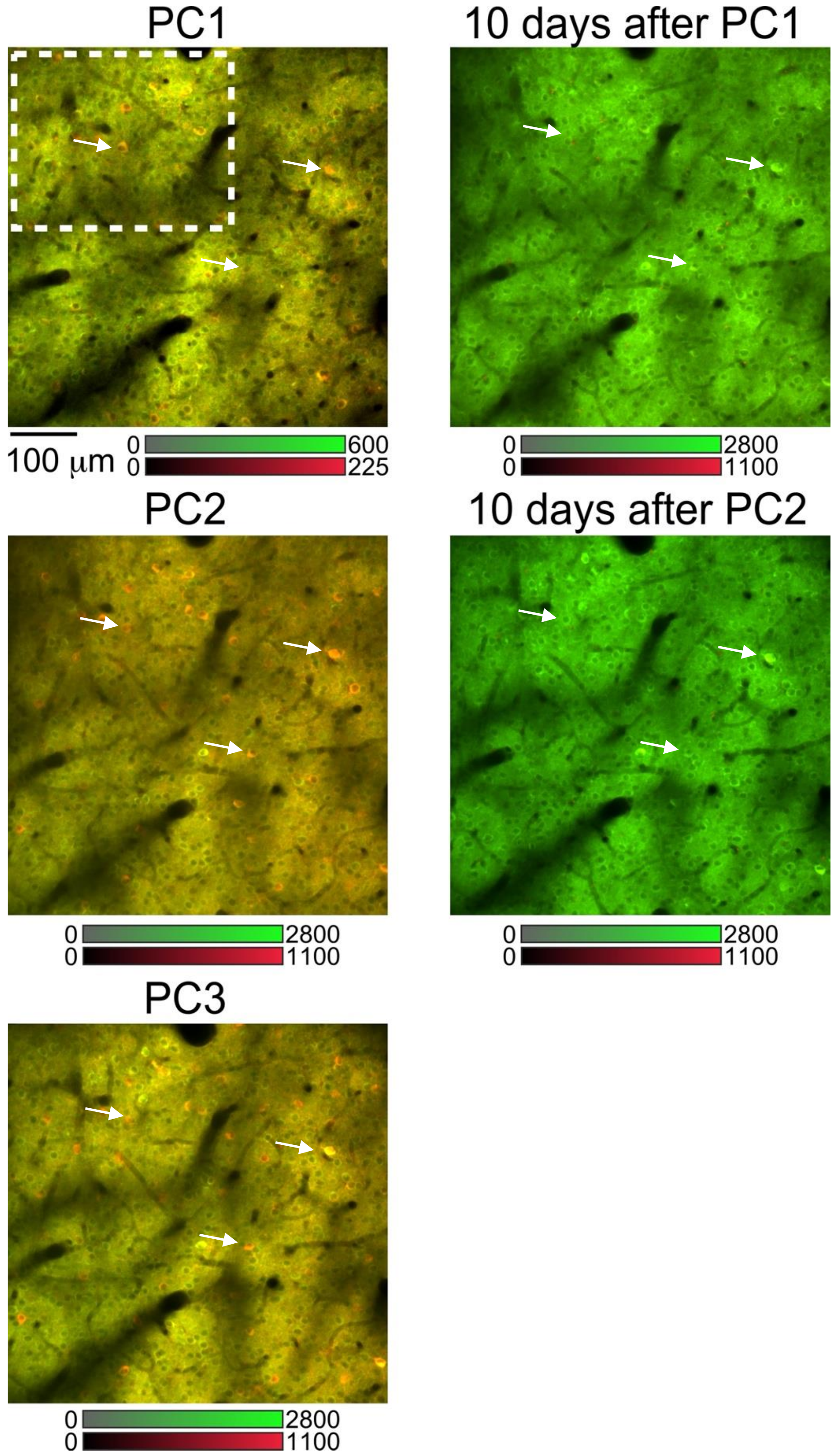

PC1
10 days after PC1
100 μm
0 600
0 225
0 2800
0 1100
PC2
10 days after PC2
0 2800
0 1100
0 2800
0 1100
PC3
0 2800
0 1100

Supplementary Figure 2. Example images of repeated PCs of the same neurons. To demonstrate that CaMPARI can be used for multiple recordings, neurons in the mouse V1 were photoconverted three times using 300 Joules/mm^2 during the presentation of a drifting grating movie (see Methods for details). The same FOV was recorded 10 days after the 1st and 2nd PCs to confirm the decay of the red signal, and then an additional PC was performed. The zoomed-in region within the dashed-line rectangle is shown in Fig. 1c. White arrowheads show three example cells across the different recording days. Repeated PC experiments were conducted in 2 mice with consistent results, no further PCs were done following the 3rd PC.

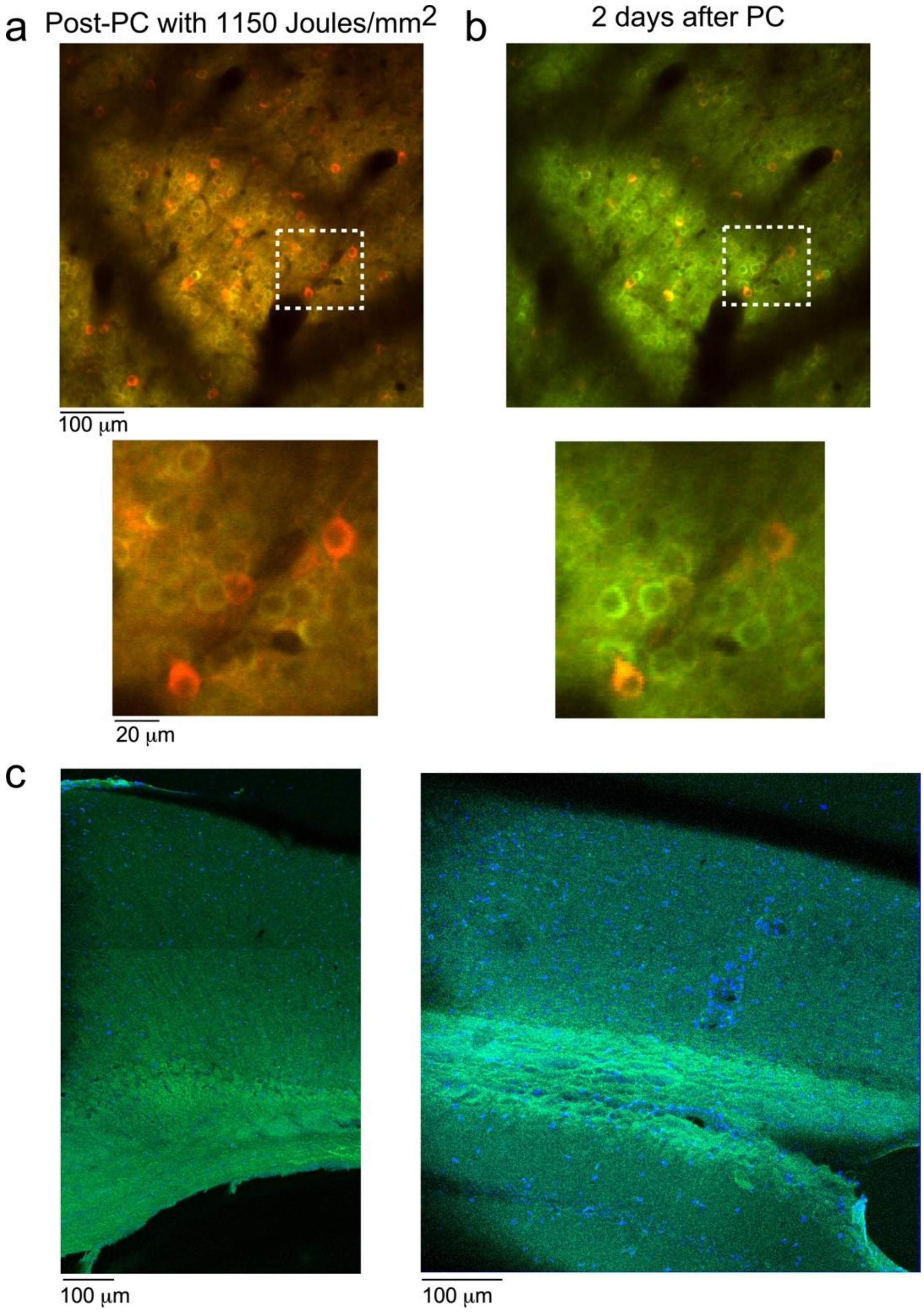

Supplementary Figure 3. No apparent damage to neurons after PC with up to 1150 Joules/mm^2. n=3 mice were photoconverted with 1150 Joules/mm^2 and perfused 2 days later, all results were consistent. **(a)** Example images of photoconverted V1 neurons shortly after PC. The area within the white rectangle (top) is magnified at the bottom. **(b)** Images of the same FOV as in **a,** 2 days after PC, showed no loss of cells. Imaging from 9 FOVs (n=3 mice) for 2 weeks after PC showed no loss of cells. **(c)**. The brain tissue was labeled with Fluoro-JadeC to monitor for cell degeneration due to heating, but no cell death was identified. Two example images show the cortex under the cranial window labeled with Fluoro-JadeC (green) and DAPI (blue).

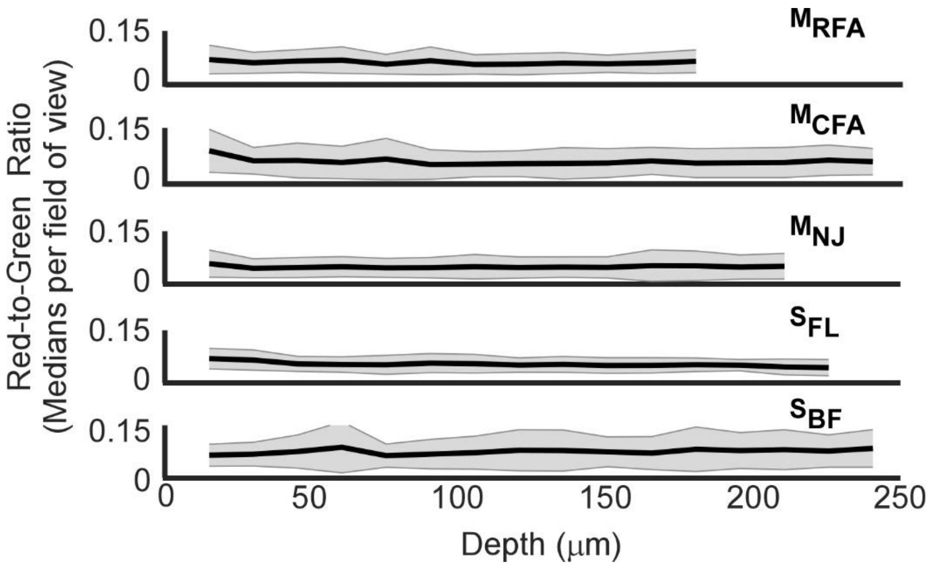

Supplementary Figure 4. No significant effect of recording depth on RGR levels was identified. We compared RGR values measured at different tissue depths across different brain areas during the NOR test. RGRs from different brain regions at different depth showed no apparent decrease (M_{RFA}**:** 4 mice, 5-51 cells/FOV, median number of cells= 17; M_{CFA}**:** 3 mice, 1-85 cells/FOV, median= 13; M_{NJ}**:** 4 mice, 5-73 cells/FOV, median= 18; S_{FL}**:** 3 mice, 4-64 cells/FOV, median= 13; S_{BF}**:** 5 mice, 2-110 cells/FOV, median= 23; shaded areas show the standard deviation and solid lines show the average across all median measurement at a specific depth).

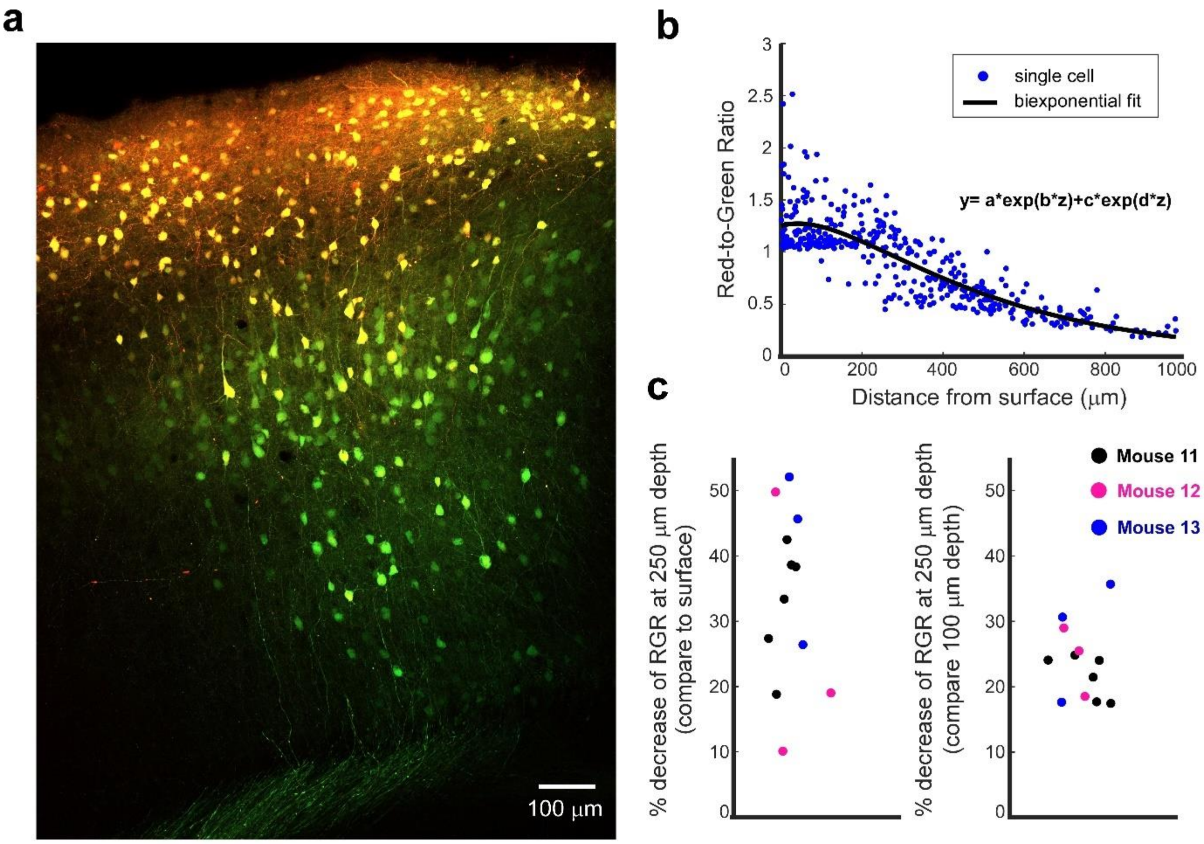

d

Mouse	FOV	biexponential coefficients			
		a	b	c	d
11	1	1.69600	-0.00129	0.00057	0.00563
	2	0.82070	-0.00096	0.42600	-0.00718
	3	1.63200	-0.00238	-0.68080	-0.00671
	4	0.80610	-0.00262	0.08222	0.00173
	5	1.13800	-0.00171	0.01156	0.00272
	6	1.08400	-0.00255	0.11060	0.00155
12	1	0.38050	-0.01986	2.09000	-0.00209
	2	2.28800	-0.00257	-1.03800	-0.00677
	3	-1.31200	-0.01152	2.39200	-0.00331
13	1	4.14100	-0.00294	0.00000	0.01014
	2	2.04800	-0.00244	0.00000	-0.00244
	3	-0.08705	-0.00858	1.68000	-0.00141

Supplementary Figure 5. Characterization of PC efficiency as a function of tissue depth. n=3 mice were injected with mEOS, a calcium-insensitive photoconvertible protein, and then illuminated with the same PC protocol used for recording visual activity and ~100 Joules/mm^2 light dose. The mice were perfused with 4% paraformaldehyde shortly after the experiment and coronal tissue sections were imaged under a confocal microscope (see an example image in **a**). **(b)** For each FOV, all identifiable cells (blue dots) were segmented and their distance from the tissue surface was measured. The

depth vs. RGR data were fit with a bi-exponential curve (black line). **(c)** Percent decreases in RGR within Layers I-III or within Layer II/III were based on the bi-exponential fit for each recorded slice and were calculated by dividing the RGR value at the bottom of Layer II/III (depth of 250 μm) by either the value on the surface (left) or by the RGR at the upper part of Layer II/III (100 μm depth, right). For most samples, the attenuation within Layer II/III was in the range of 20-30%. **(d)** A summary of the bi-exponential fit parameters for all recorded samples from n=3 mice.

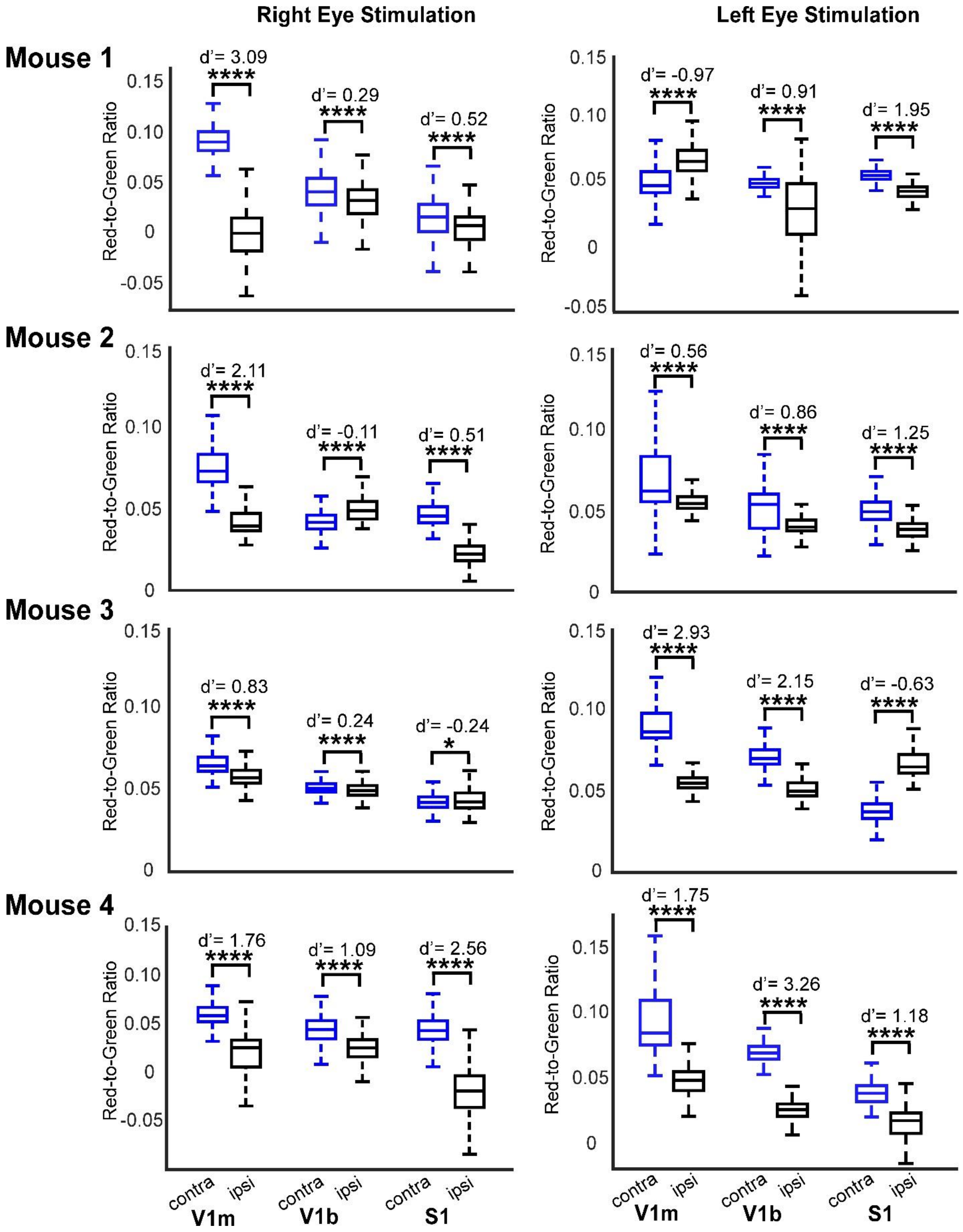
Right Eye Stimulation
Left Eye Stimulation
Mouse 1
Mouse 2
Mouse 3
Mouse 4
Red-to-Green Ratio
d'= 3.09
d'= 0.29
d'= 0.52
d'= -0.97
d'= 0.91
d'= 1.95
d'= 2.11
d'= -0.11
d'= 0.51
d'= 0.56
d'= 0.86
d'= 1.25
d'= 0.83
d'= 0.24
d'= -0.24
d'= 2.93
d'= 2.15
d'= -0.63
d'= 1.76
d'= 1.09
d'= 2.56
d'= 1.75
d'= 3.26
d'= 1.18
contra
ipsi
V1m
V1b
S1

Supplementary Figure 6. Single-mouse data of visual-evoked responses in each brain region show increased activity in contralateral V1 regions, as recorded with CaMPARI2. Summary of RGR values for n=4 recorded mice. Each mouse was recorded twice for detecting activity patterns over 6 brain regions in 2 hemispheres after stimulation of either the right or the left eye. For most recordings, the activities of contralateral V1m and V1b regions were higher than their ipsilateral counterparts, with significant increases in the median activity level. Changes across contralateral and ipsilateral S1 activity were significant for single recordings (when all recorded cells are used for the statistical comparison), but not when comparing median RGR values across recordings. Data from mouse 4 left eye is also presented in Fig. 1d, and the summary of median activity levels from all mice is shown in Fig. 1e of the manuscript. Values of the sensitivity index (d') and significance of comparison across the same brain regions in the contralateral and ipsilateral hemispheres are shown (Wilcoxon Ranksum Tests, **** $p<0.0001$, * $p<0.05$; Mouse 1: 107-1219 cells/region, median= 429; Mouse 2: 81-456 cells/region, median= 324; Mouse 3: 214-1455 cells/region, median=868; Mouse 4: 111-350 cells/region; median=249; see also source data file and raw data: 10.6084/m9.figshare.24065970).

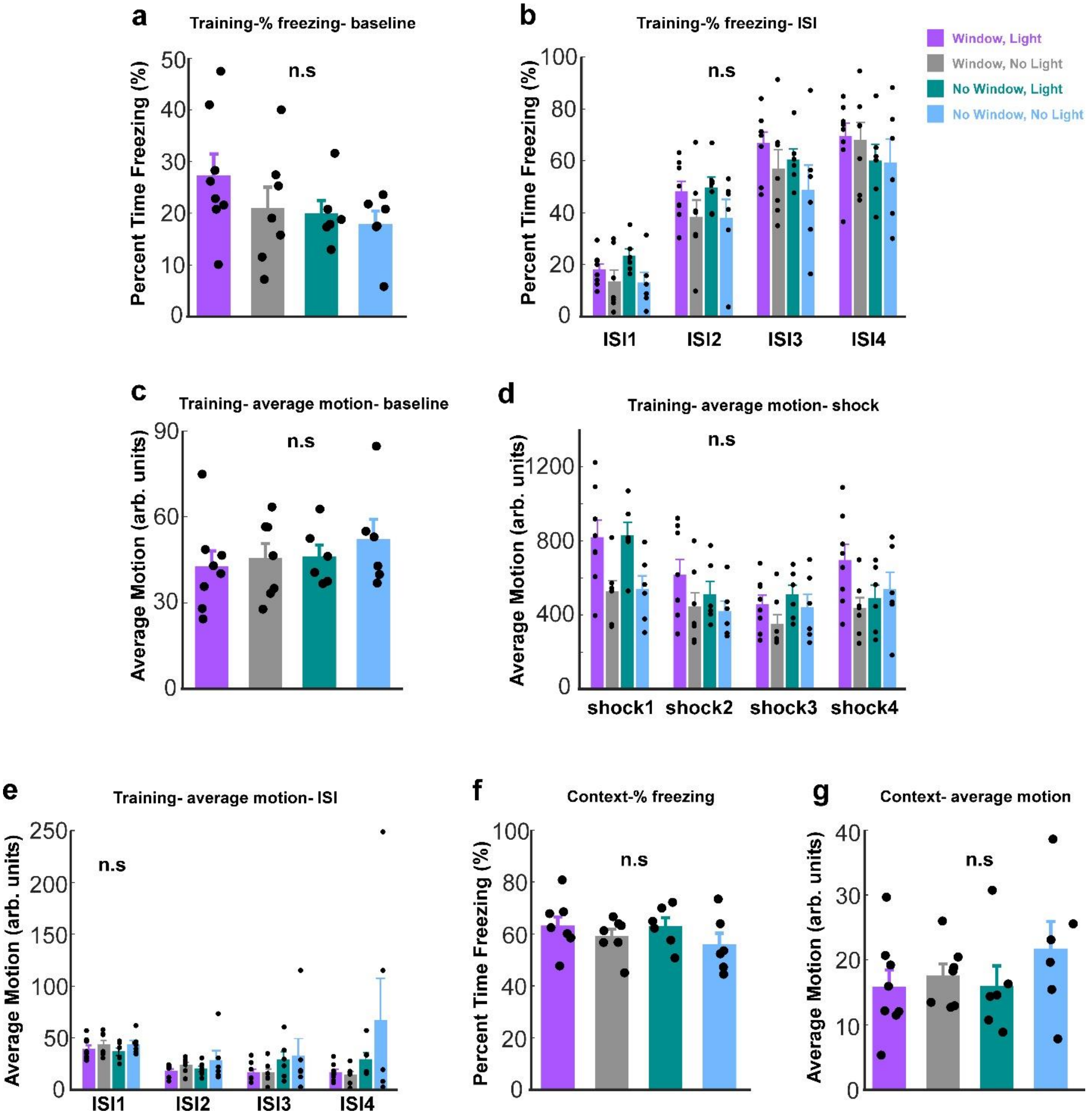

Supplementary Figure 7. No apparent effect of cranial window implantation and PC light illumination on mouse performance in the fear conditioning (FC) task. (a-g) C57Bl6/J male mice (n=15) were implanted with cranial windows and randomly divided into 2 groups that were tested for the FC task with PC light illumination (n=8 mice; Window, Light group) or without it (n=7 mice; Window, No Light group). An additional 12 naïve C57Bl6/J male mice without any intervention were randomly divided into 2 equal groups and were also tested with and without PC light illumination (see Methods for details). (**a-e**) Quantification of freezing and average motion parameters during the FC

task training, shock, and ISI phases. (**f-g**) Quantification of percent freezing and average motion during the contextual fear memory test. No significant differences were found among the groups (one-way ANOVA test). Bars show the mean values, error bars show the standard error, and dots show data from individual mice.

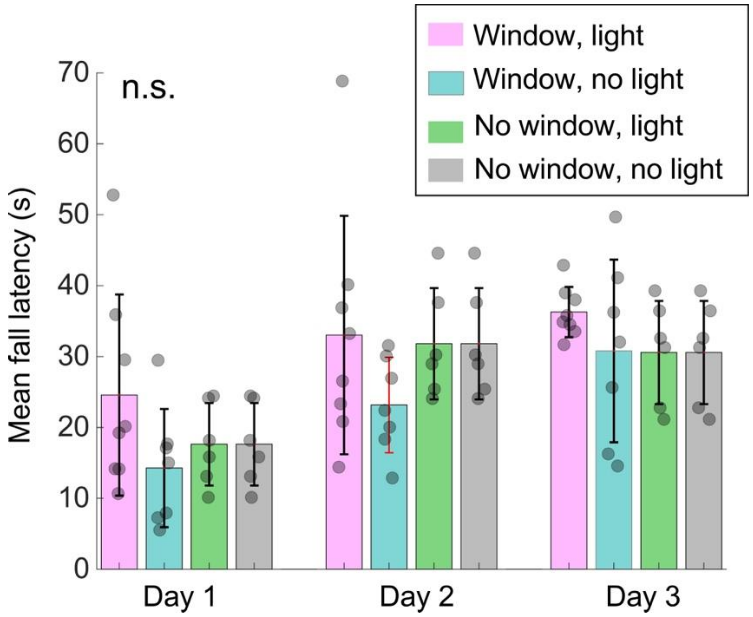

Supplementary Figure 8. No apparent effect of cranial window implantation or PC light illumination on mouse performance in the rotarod (RR) task. The same mice described in Supp. Fig. 7 were tested in the RR test. We quantified the mean fall latency for each mouse (black dots) for each recording day. There was no significant difference among the 4 groups for each day (one-way ANOVA test among the 4 groups for each day with p=0.239, 0.341, and 0.48 for days 1, 2, and 3, respectively). Bars show the mean values and error bars show the standard deviation.

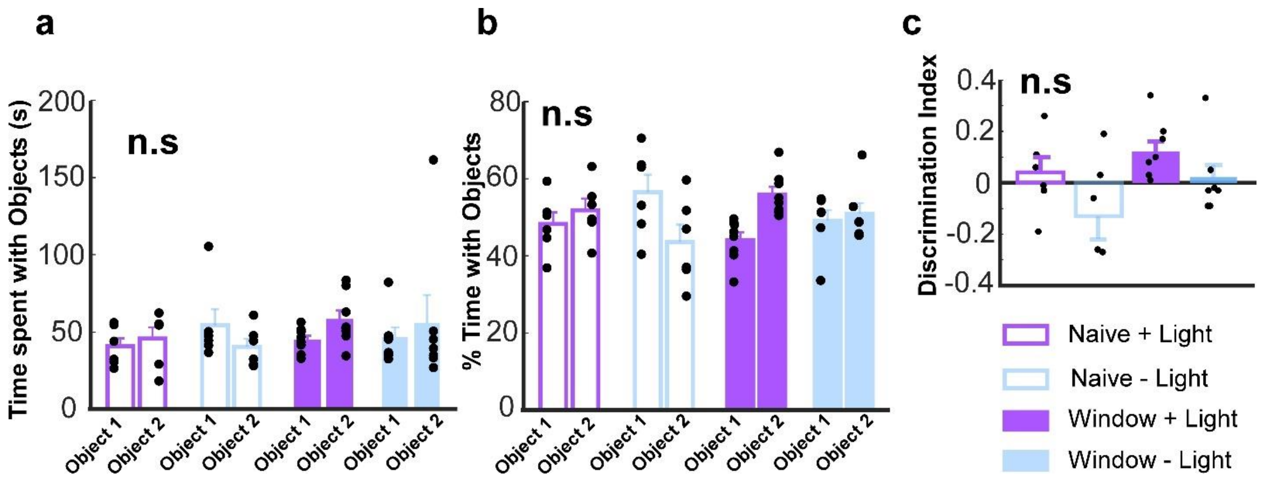

Supplementary Figure 9. No apparent effect of cranial window implantation or PC light illumination on mouse performance in the novel object recognition (NOR) task. The same mice described in Supp. Figs. 7-8 were also tested in the NOR task. (**a**) Quantification of the time and (**b**) the percent of time spent with each object (object 1 – known, object 2 – novel) as well as the (**c**) discrimination index. Although these mice showed a lower preference towards the novel object than other tested mice (see Fig. 4e for an example), there was no significant effect of the cranial window and/or PC light illumination on mouse preference (one-way ANOVA test). Bars show the mean values, error bars show the standard error, and dots show data from individual mice.

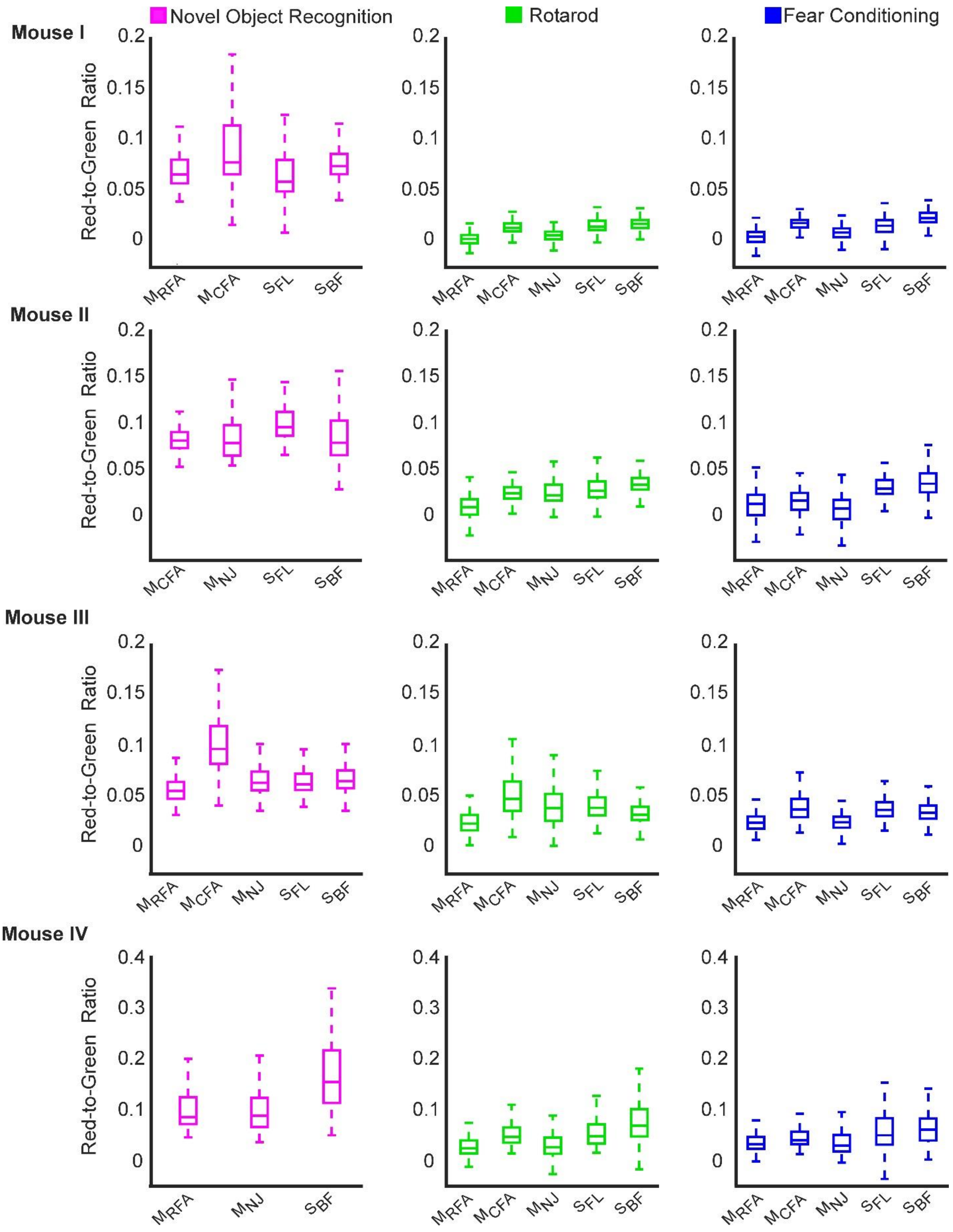

Novel Object Recognition
Rotarod
Fear Conditioning
Mouse I
Mouse II
Mouse III
Mouse IV
Red-to-Green Ratio
MRFA
MCFA
MNJ
SFL
SBF

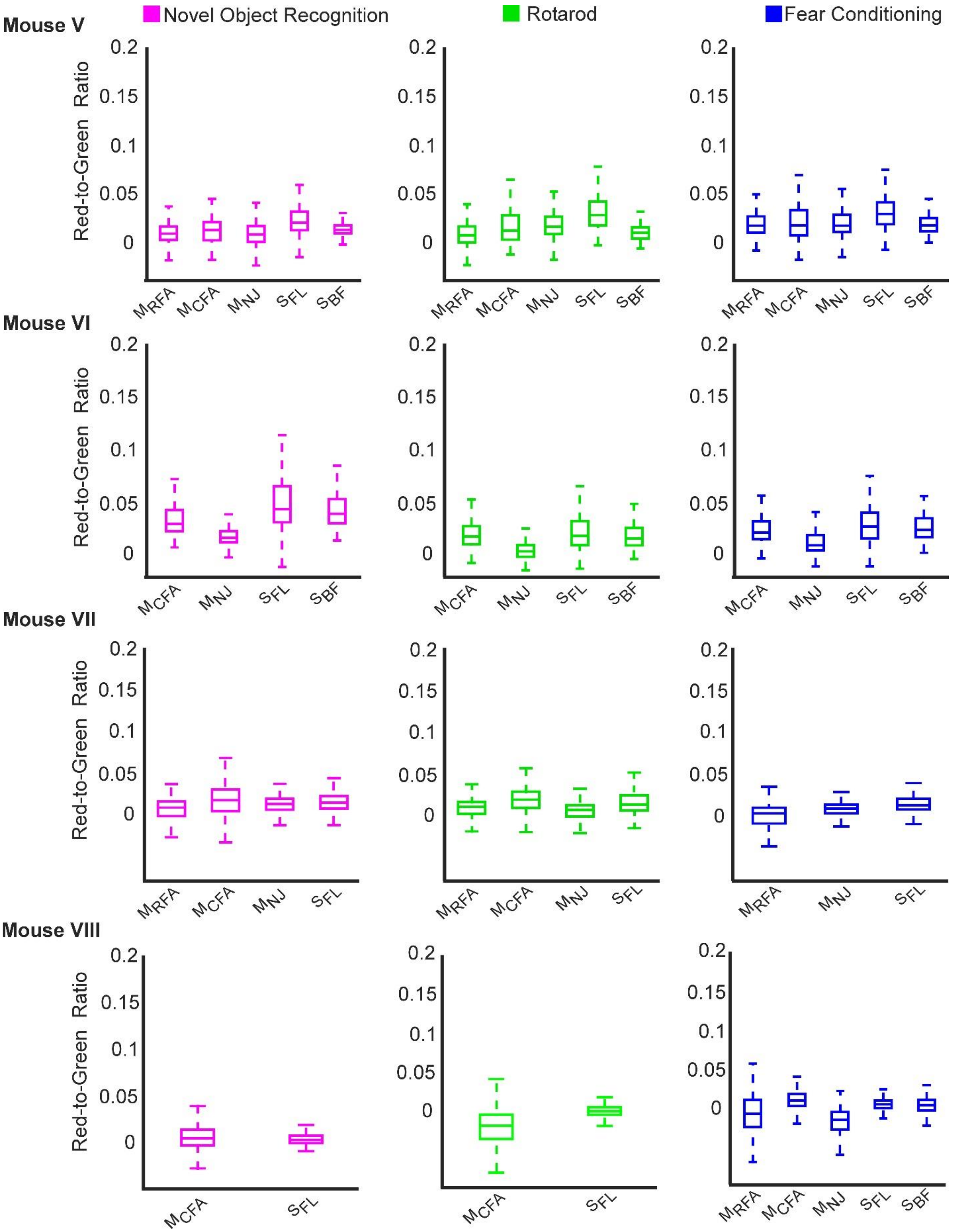

Novel Object Recognition
Rotarod
Fear Conditioning
Mouse V
Mouse VI
Mouse VII
Mouse VIII
Red-to-Green Ratio
0.2
0.15
0.1
0.05
0
MRFA
MCFA
MNJ
SFL
SBF

Supplementary Figure 10. Differential levels of brain activity in individual freely-moving mice during NOR, RR, and FC tasks. Summary of RGR from all recorded brain regions of all recorded freely-moving mice (n=8) while performing three different behavioral and cognitive tasks (Mouse I: 95-905 cells/region, median=441; Mouse II:42-763 cells/region, median=144; Mouse III: 166-1315 cells/region, median=597; Mouse IV: 89-559 cells/region, median=177; Mouse V: 94-906 cells/region, median=271; Mouse VI: 116-550 cells/region, median=339; Mouse VII: 200-684, median=329; Mouse VIII: 128-432, median=327), Data from mouse III is presented in Fig. 2d, and the median values from all mice were used in Fig. 2e-g. Note that not all brain regions were recorded in all sessions, due to changes in the recording quality (see Methods; access to raw data: 10.6084/m9.figshare.24065970).

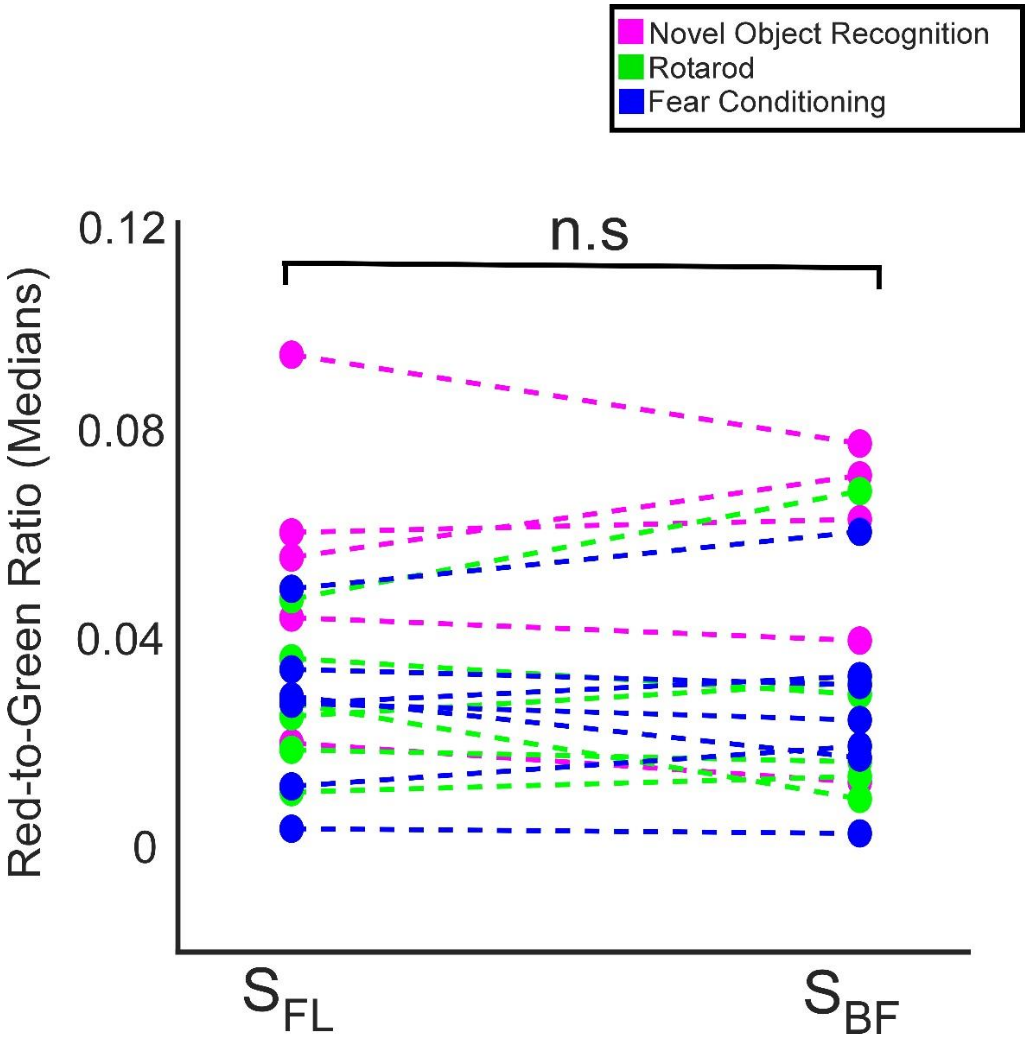

Supplementary Figure 11. No significant activity changes across the recorded somatosensory regions of freely-moving mice (n=7). Comparison of S_{FL} and S_{BF} during the three tested tasks showed no significant changes (n.s, p=0.946, paired t-test, 42-1315 cells/region, median= 285).

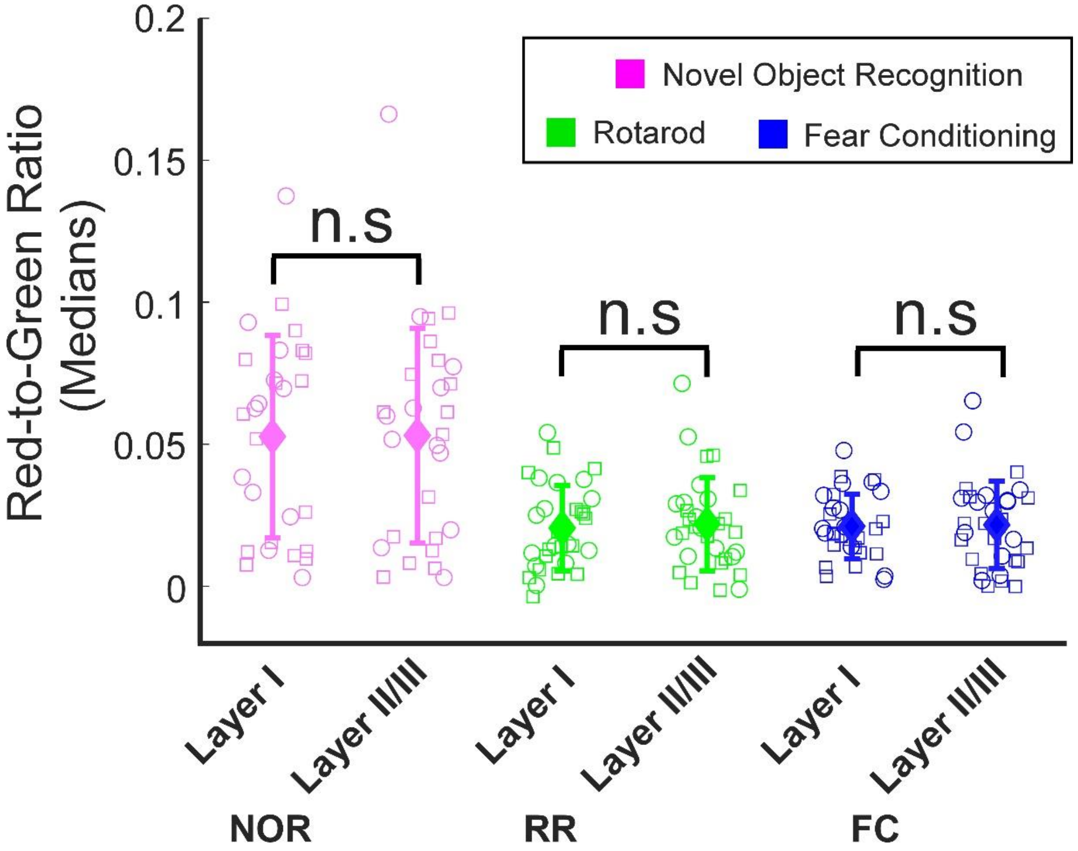

Supplementary Figure 12. Comparison of activity in layer I and layer II/III cells showed similar activity levels. We compared the RGR across simultaneously-recorded layer I and layer II/III neurons during the NOR, RR, and FC tasks. Activity levels showed similar values and no significant changes were found (paired t-test, data from 34 recorded regions from n=8 mice; **NOR:** Layer I, 9-318 cells/region, median= 76; Layer II/III, 24-765 cells/region, median = 226; p value = 0.83, **RR:** Layer I, 14-183 cells/region, median= 76; Layer II/III, 41-693 cells/region, median = 195; p value = 0.22, **FC:** Layer I, 31-167 cells/region, median= 63; Layer II/III, 47-1189 cells/region, median = 253; p value = 0.73; bold diamonds and error bars show mean ± standard deviation)

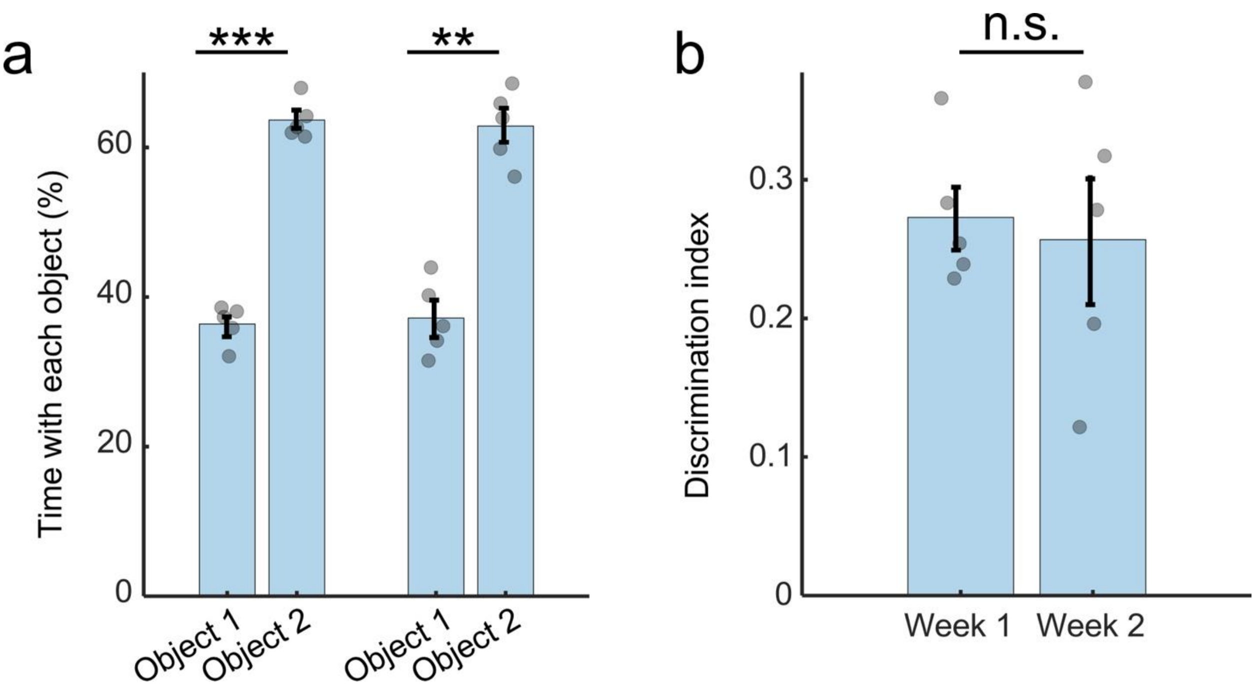

Supplementary Figure 13. No apparent difference in the performance of mice in the NOR task over two consecutive weeks. The same n=5 mice were tested for two consecutive weeks in the NOR task, with two new objects for every week. (**a**) The mice spent significantly more time with the novel object for both weeks (p=0.0003 and 0.004 for weeks 1 and 2, respectively, paired t-tests). (**b**) The discrimination indices for both weeks were similar (no significant changes were detected across the two weeks, p=0.78, paired t-test; Each bar graph shows mean ± standard deviation).

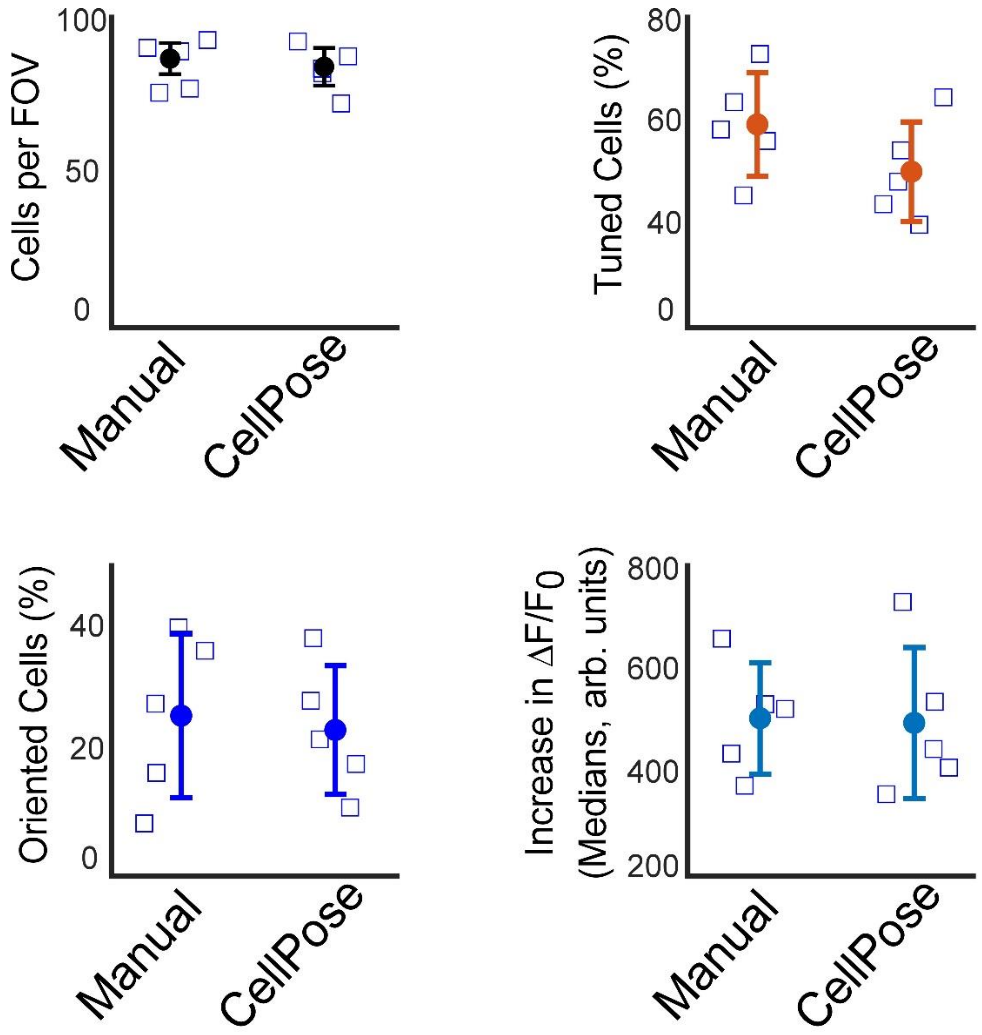

Supplementary Figure 14. Comparing CellPose vs. manual cell segmentation methods and their effects on data analysis. We compared a semi-automated MATLAB code for identifying cells (from Chen et al., *Nature*, 2013) where a user manually identifies cells and the software detects the soma boundaries (manual method) with the CellPose algorithm (Stringer et al., *Nature* Methods, 2021), which is designed to identify soma location from images. The two methods were used to identify cells in the same dataset (n=1 mouse, 5 FOVs) recorded from V1 neurons labeled with jGCaMP7s. We found no significant differences between the two methods for the number of segmented cells, fractions of tuned or oriented cells, or the identified increases of the fluorescence during visual stimulation (bold circles and error bars show mean ± standard deviation).

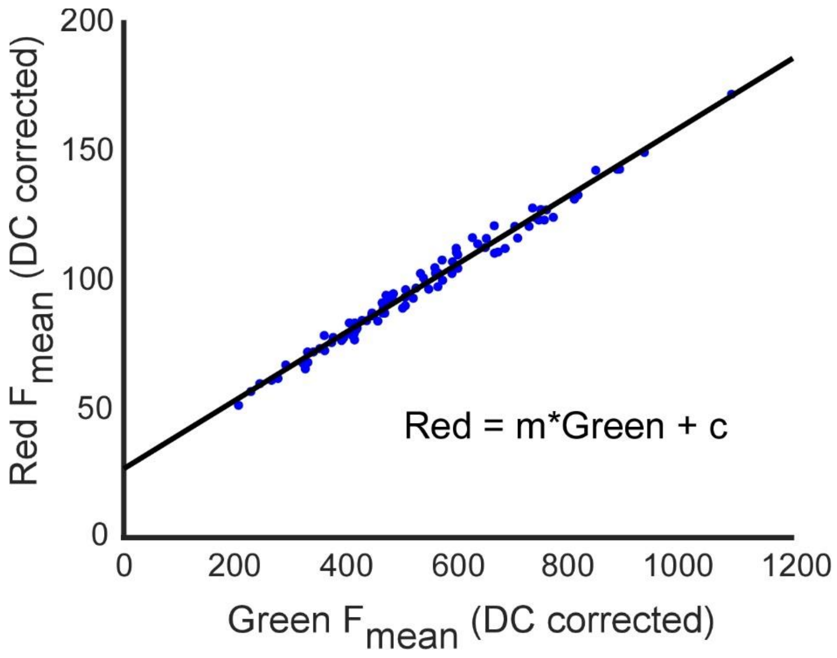

Supplementary Figure 15. Calculation of RGR values from raw fluorescence data. Example somatic fluorescence signals from non-photoconverted neurons (after dark current subtraction) showed a linear relationship between the red and green fluorescence signals due to penetration of the green CaMPARI signal into the red channel. To correct for this contamination, we fit the acquired data with a 1st-order polynomial, to calculate the slope and the intercept (m and c, respectively; see equation). The green-to-red contamination factor was calculated by averaging m values from multiple recordings and was specific for the detection system characteristics, like the detection filter set that was used to separate the green and red channels, the PMT detectors and their gain, and the detection optics. In addition, the line intercept (c) usually had a small positive value, presumably due to a weak autofluorescence in the red channel, which was subtracted as well. m values were calculated separately for different experiments described in Figs. 1, 2 and 3, since they were conducted using either different microscopes, different filter sets, or with different detection optics.

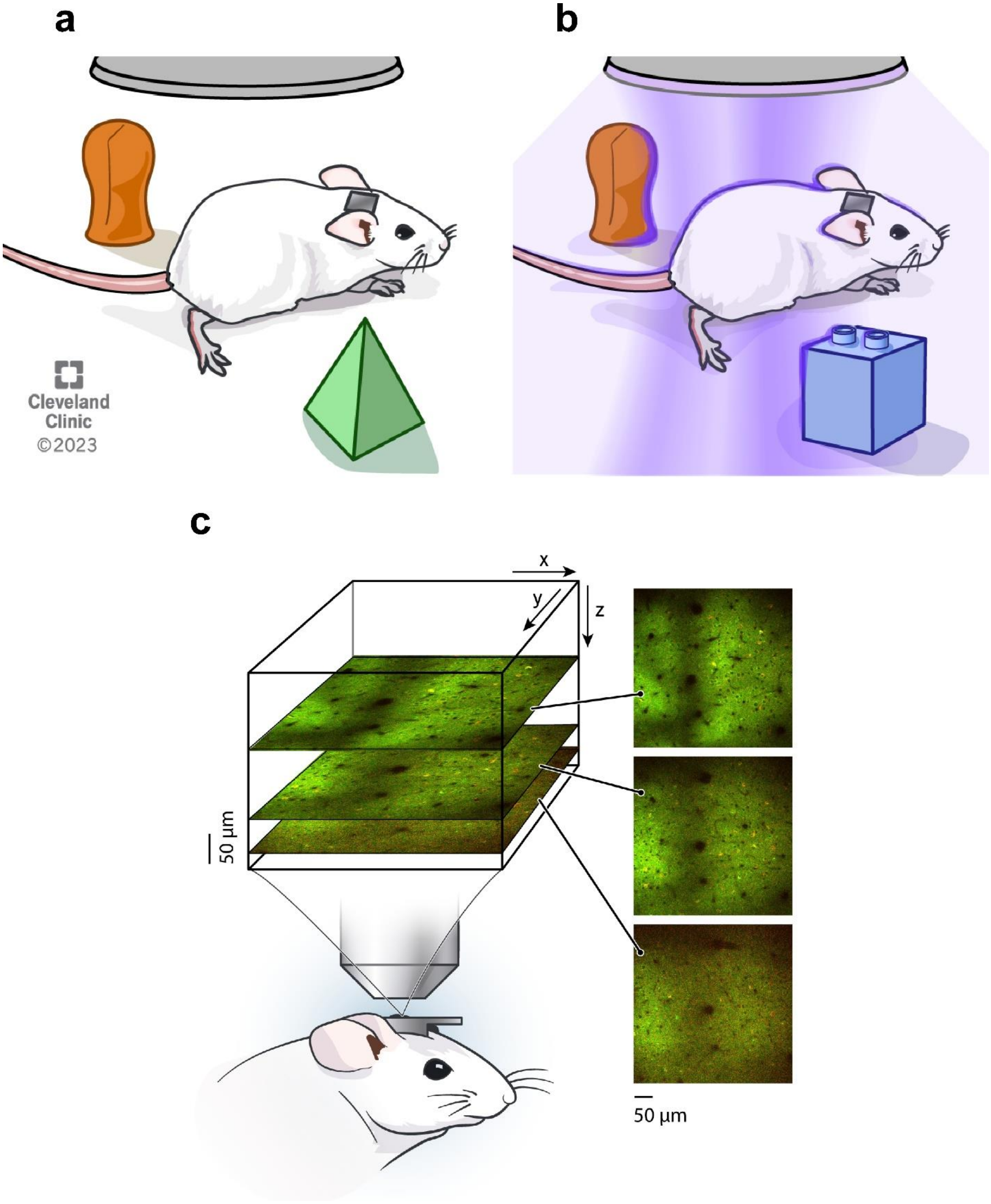

Supplementary Figure 16. Summary of the method. We present a method for recording large-scale, volumetric brain activity from freely-moving mice with single-cell resolution and no mechanical restriction to the animals during recording. **(a)** The animal is trained in the arena on a specific task. **(b)** Once the animal is ready, the PC light is

turned on and the brain activity is recorded. Once the PC light is turned off, the activity signal (RGR) remains imprinted for approximately 24 hours. **(c)** Activity readout using TPLSM from all identifiable neurons, down to a depth of 300μm, can conducted on a later time. Reprinted with permission, Cleveland Clinic Foundation ©2023. All Rights Reserved.
