# Supplementary material for: Large-scale recording of neuronal activity in freely-moving mice at cellular resolution
Source: Nat Commun. 2023 Oct 12;14:6399. doi: 10.1038/s41467-023-42083-y (PMC10570384; doi:10.1038/s41467-023-42083-y)
Supplement: Supplementary file 2 — Description of Additional Supplementary Files [file 41467_2023_42083_MOESM2_ESM.pdf]

Description of Additional Supplementary Files

File name: Supplementary Movie 1

Description: Recording from a freely-moving mouse during the novel object recognition testing. The PC light source was located on top of the enclosed arena and illuminated it in its entirety (the same vertical distance from the arena was maintained across all tasks to provide identical PC conditions), and the mouse was free to move inside it without any mechanical device attached to it.

File name: Supplementary Movie 2

Description: Recording from a freely-moving mouse during the rotarod task. Similarly to supplementary movie 1, the light source illuminated the entire testing region, and the mouse movement was not restricted within it.

File name: Supplementary Movie 3

Description: Recording of a freely-moving mouse during the contextual fear conditioning test. Mice were trained to associate an aversive stimulus with the environment (context). Activity recording was conducted when the mouse was put in the same environment as during the training but without the aversive stimulus.

File name: Supplementary Movie 4

Description: Example data showing *in vivo* volumetric CaMPARI recording with cellular resolution from the surface of the brain down to the bottom of layer II/III (~330 μm). Note that small tissue movements were due to animal breathing during the recording.
