# Supplementary material for: Large-scale recording of neuronal activity in freely-moving mice at cellular resolution
Source: Nat Commun. 2023 Oct 12;14:6399. doi: 10.1038/s41467-023-42083-y (PMC10570384; doi:10.1038/s41467-023-42083-y)
Supplement: Supplementary file 7 — Reporting Summary [file 41467_2023_42083_MOESM7_ESM.pdf]

nature portfolio

Corresponding author(s): Hod Dana

Last updated by author(s): 09/01/2023

Reporting Summary

Nature Portfolio wishes to improve the reproducibility of the work that we publish. This form provides structure for consistency and transparency in reporting. For further information on Nature Portfolio policies, see our Editorial Policies and the Editorial Policy Checklist.

Statistics

For all statistical analyses, confirm that the following items are present in the figure legend, table legend, main text, or Methods section.

n/a	Confirmed	
☐	☒	The exact sample size (*n*) for each experimental group/condition, given as a discrete number and unit of measurement
☐	☒	A statement on whether measurements were taken from distinct samples or whether the same sample was measured repeatedly
☐	☒	The statistical test(s) used AND whether they are one- or two-sided<br>*Only common tests should be described solely by name; describe more complex techniques in the Methods section.*
☒	☐	A description of all covariates tested
☐	☒	A description of any assumptions or corrections, such as tests of normality and adjustment for multiple comparisons
☐	☒	A full description of the statistical parameters including central tendency (e.g. means) or other basic estimates (e.g. regression coefficient) AND variation (e.g. standard deviation) or associated estimates of uncertainty (e.g. confidence intervals)
☐	☒	For null hypothesis testing, the test statistic (e.g. *F*, *t*, *r*) with confidence intervals, effect sizes, degrees of freedom and *P* value noted<br>*Give P values as exact values whenever suitable.*
☒	☐	For Bayesian analysis, information on the choice of priors and Markov chain Monte Carlo settings
☒	☐	For hierarchical and complex designs, identification of the appropriate level for tests and full reporting of outcomes
☒	☐	Estimates of effect sizes (e.g. Cohen's *d*, Pearson's *r*), indicating how they were calculated

Our web collection on statistics for biologists contains articles on many of the points above.

Software and code

Policy information about availability of computer code

Data collection	ThorImage versions 4 and 4.1 (Thorlabs); ZEN Core 3.6 (Zeiss).
Data analysis	CellPose (Stringer et al., Nature Methods, 2021); Matlab R2022b; Custom Matlab scripts were used for data analysis and were deposited in [add info here]

For manuscripts utilizing custom algorithms or software that are central to the research but not yet described in published literature, software must be made available to editors and reviewers. We strongly encourage code deposition in a community repository (e.g. GitHub). See the Nature Portfolio guidelines for submitting code & software for further information.

Data

Policy information about availability of data

All manuscripts must include a data availability statement. This statement should provide the following information, where applicable:

- Accession codes, unique identifiers, or web links for publicly available datasets
- A description of any restrictions on data availability
- For clinical datasets or third party data, please ensure that the statement adheres to our policy

All relevant data are included as part of the supplementary information or have been deposited in the FigShare database under accession code 10.6084/m9.figshare.24065970

Research involving human participants, their data, or biological material

Policy information about studies with human participants or human data. See also policy information about sex, gender (identity/presentation), and sexual orientation and race, ethnicity and racism.

Reporting on sex and gender	N/A
Reporting on race, ethnicity, or other socially relevant groupings	N/A
Population characteristics	N/A
Recruitment	N/A
Ethics oversight	N/A

Note that full information on the approval of the study protocol must also be provided in the manuscript.

Field-specific reporting

Please select the one below that is the best fit for your research. If you are not sure, read the appropriate sections before making your selection.

☒ Life sciences ☐ Behavioural & social sciences ☐ Ecological, evolutionary & environmental sciences

For a reference copy of the document with all sections, see nature.com/documents/nr-reporting-summary-flat.pdf

Life sciences study design

All studies must disclose on these points even when the disclosure is negative.

Sample size	Sample size was determined by running a power analysis based on preliminary recordings with a small cohort of CaMPARI-expressing mice, before the experiments described in the manuscript were started.
Data exclusions	Data exclusion was based on the quality of the fluorescence signal and in one experiment, on animal behavior that biased the analysis.
Replication	Replications of the NOR test was done with n=5 mice, as well as testing of the decay of the red signal after photoconversion (2 mice), and repeated photoconversions for testing for photodamage (3 mice). All replications were successful and showed consistent results.
Randomization	Mice were randomly allocated to groups. The order of the behavioral tests was not random due to animal welfare considerations.
Blinding	Blinding was not relevant, since this paper develops a new method and didn't compare the same experimental procedures on different groups. There was only one CaMPARI group, so blinding could not hide it.

Reporting for specific materials, systems and methods

We require information from authors about some types of materials, experimental systems and methods used in many studies. Here, indicate whether each material, system or method listed is relevant to your study. If you are not sure if a list item applies to your research, read the appropriate section before selecting a response.

Materials & experimental systems

n/a	Involved in the study	
☒	☐	Antibodies
☒	☐	Eukaryotic cell lines
☒	☐	Palaeontology and archaeology
☐	☒	Animals and other organisms
☒	☐	Clinical data
☒	☐	Dual use research of concern
☒	☐	Plants

Methods

n/a	Involved in the study	
☒	☐	ChIP-seq
☒	☐	Flow cytometry
☒	☐	MRI-based neuroimaging

Animals and other research organisms

Policy information about studies involving animals; ARRIVE guidelines recommended for reporting animal research, and Sex and Gender in Research

Laboratory animals	C57BL6/J. EMX-1, and B6 PV-CRE mice , males and females, 8-12 weeks old at the beginning of the study. All mice were held in an AAALAC-approved vivarium facility. The vivarium was maintained at 20-22°C with 30-70% humidity and a 12-hour light/12-hour dark cycle. The experiments were conducted during the light time.
Wild animals	No wild animals were used in the study.
Reporting on sex	Both males and females were used in the study [20 females and 47 males]
Field-collected samples	No field-collected samples were used in the study.
Ethics oversight	All experimental and surgical procedures were performed following the set guidelines and protocols approved by the Lerner Research Institute (LRI) and Oregon Health & Science University (OHSU) Institutional Animal Care and Use Committees (IACUCs) and Institutional Biosafety Committees (IBCs) .

Note that full information on the approval of the study protocol must also be provided in the manuscript.
